# Ligand‐Independent Activation of Notch1 by Cathepsin L Induces CUX1/p16^INK4a^ ‐Dependent Endothelial Senescence Associated With Atherosclerosis

**DOI:** 10.1111/acel.70563

**Published:** 2026-05-28

**Authors:** Yuwei Wu, Lili Lu, Shaoyang Yan, Zewen Du, Danli Jiang, Ting Wu, Qichao Wu, Jie Liu, Johny Ebin, Wei Sun, Partha Dutta, Jay Xiaojun Tan, Jonathan K. Alder, Gang Li

**Affiliations:** ^1^ Aging Institute University of Pittsburgh Pittsburgh Pennsylvania USA; ^2^ Department of Cardiology, Third Xiangya Hospital Central South University Changsha China; ^3^ Center for Pulmonary Vascular Biology and Medicine, Pittsburgh Heart, Lung, Blood, and Vascular Medicine Institute University of Pittsburgh School of Medicine and University of Pittsburgh Medical Center Pittsburgh Pennsylvania USA; ^4^ Department of Cell Biology University of Pittsburgh School of Medicine Pittsburgh Pennsylvania USA; ^5^ Division of Pulmonary, Allergy, and Critical Care Medicine, Dorothy P. and Richard P. Simmons Center for Interstitial Lung Disease University of Pittsburgh Pittsburgh Pennsylvania USA; ^6^ Division of Cardiology, Department of Medicine University of Pittsburgh Medical Center Pittsburgh Pennsylvania USA

## Abstract

Our post‐GWAS functional analysis revealed that cathepsin L (CTSL) is an upstream regulator of CUX1, and it induces p16^INK4a^‐dependent and atherosclerosis‐associated senescence by indirectly activating CUX1 transcription in a process that requires its proteolytic activity. This suggests an unidentified transcription regulator between CTSL and CUX1, and CTSL‐mediated cleavage of this regulator could transcribe CUX1, inducing senescence. Here, in search of this transcriptional regulator, we discovered that Notch1 is a substrate of CTSL, and CTSL can proteolytically activate Notch1 in a ligand‐independent fashion, liberating NICD. NICD, after complexing with RBPJ in the nuclei, induces CUX1/p16^INK4a^‐dependent senescence. Consistently, an upregulation of both CTSL and NICD, along with elevated cellular senescence in the plaques isolated from patients with atherosclerosis, was observed. In addition, we showed that endothelial deletion of CUX1 in the *atherosclerosis‐prone* ApoE^−/−^ mice blocks high‐fat diet‐induced senescence throughout the entire plaques, and these ApoE^−/−^ mice exhibit similar phenotypes as the atherosclerosis‐prone models with CTSL and Notch1/RBPJ inactivation including attenuated atherosclerotic lesion, intact and well‐organized elastin fibers, and reduced macrophage content of plaque. This further supports our findings that both CTSL and Notch1/RBPJ are upstream regulators of CUX1, regulating senescence. Thus, while our studies identify a non‐canonical Notch1 pathway that can be activated by CTSL in a ligand‐independent fashion to induce senescence, our findings also reveal a role of senescence in the development of atherosclerosis. This provides new insight into developing drugs aimed to target cellular senescence for atherosclerosis.

## Graphical abstract

CTSL activates Notch1/RBPJ in a ligand‐independent fashion, inducing CUX1/p16^INK4a^‐dependent endothelial senescence associated with atherosclerosis.

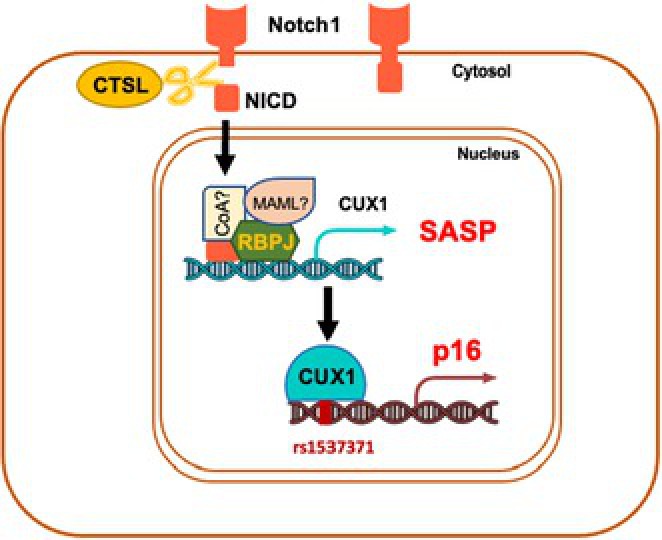

## 1 Introduction

Cellular senescence is defined as irreversible cell cycle arrest, often accompanied by an enlarged and flattened cellular morphology, which could disrupt tissue homeostasis (Zhang et al. 2022). In association with this arrest, senescent cells also secrete multiple proinflammatory molecules such as cytokines IL‐6 and IL‐1β, express cell adhesion molecules such as ICAM‐1 and VCAM‐1, and produce matrix metalloprotease such as elastase and collagenase, which collectively is known as the senescence‐associated secretory phenotype (SASP) (Gorgoulis et al. 2019). Based on the initiating trigger, cellular senescence can be classified as either replicative senescence (RS) or stress‐induced premature senescence (SIPS). Both types of senescence can be mediated through the p16^INK4a^ pathway (Gorgoulis et al. 2019; Baker et al. 2016; Khosla et al. 2020; Mijit et al. 2020), but how various upstream signals are transduced to ultimately activate p16^INK4a^ is incompletely characterized. Both RS and SIPS are now recognized as primary senescence. In contrast, secondary senescence occurs when primary senescent cells trigger senescence in neighboring cells via either juxtacrine or paracrine signaling (Admasu et al. 2021).

Recently, based on our post‐GWAS functional studies on the *CDKN2A/B* locus, a locus that carries p16^INK4a^, a known activator of cellular senescence and an important contributor to the pathogenesis of various age‐related diseases (Baker et al. 2016, 2011; Dang et al. 2020; Hickson et al. 2019), we demonstrated that cut‐like homeobox 1 (CUX1) is a major activator of p16^INK4a^, regulating both RS and SIPS by modulating p16^INK4a^ expression via its specific binding to a functional SNP rs1537371 on the *CDKN2A/B* locus (Jiang et al. 2022; Wu et al. 2023), and manipulation of p53 expression do not alter the CUX1 expression and p16^INK4a^‐dependent senescence (Jiang et al. 2022). In addition, we also discovered that cathepsin L (CTSL) regulates both RS and SIPS at least in human endothelila cells (ECs) and vascular smooth muscle cells (VSMCs) by activating *CUX1* transcription indirectly in a process that requires the proteolytic activity of CTSL (Wu et al. 2024). This finding suggests that there is at least an unknown transcription regulator between CTSL and CUX1, and cleavage of this factor by CTSL can activate CUX1 transcription, inducing p16^INK4a^‐dependent cellular senescence.

Notch1 is a single‐pass transmembrane receptor involved in inflammation and oxidative stress (Cai et al. 2016). In the canonical Notch1 pathway, Notch1 can be proteolytically activated by γ‐secretase in a ligand‐dependent fashion, liberating the Notch Intracellular Domain (NICD). Once NICD is translocated into the nucleus, it forms a transcriptional complex with the recombination signal binding protein for immunoglobulin kappa J (RBPJ), regulating the transcription of multiple downstream target genes (Kopan and Ilagan 2009). Notch1 can also be activated in a ligand‐independent mechanism (Palmer and Deng 2015). Increasing evidence demonstrates that Notch1 is involved in regulating cellular senescence, especially in secondary senescence (Hoare et al. 2016; Kagawa et al. 2015; Liu et al. 2012; Teo et al. 2019). However, how Notch1 is activated during cellular senescence and what the Notch1 target genes are in regulating cellular senescence are largely unknown.

Atherosclerosis has been recognized as an age‐related disease since its clinical incidence rises exponentially with age (Head et al. 2017). GWAS have identified a strong association of the *CDKN2A/B* locus with atherosclerosis and its complications (Helgadottir et al. 2007; Wellcome Trust Case Control, C 2007; McPherson et al. 2007), suggesting that cellular senescence could play a crucial role in this disease. Consistent with the reported roles of CTSL and Notch1/RBPJ in regulating cellular senescence as abovementioned, attenuation of atherosclerotic lesion with intact and well‐organized elastin fibers and reduced macrophage content in plaque were observed in the mouse atherosclerosis models with CTSL and Notch1/RBPJ inactivation (Kitamoto et al. 2007; Qin et al. 2016; Binesh et al. 2018; Nus et al. 2016). This suggests that both CTSL and Notch1/RBPJ could induce atherosclerosis by activating cellular senescence. However, even though we have reported that CUX1 can activate p16^INK4a^‐dependent cellular senescence through the atherosclerosis‐associated fSNP rs1537371 on the *CDKN2A/B* locus (Jiang et al. 2022; Wu et al. 2023), and elevated expression of CUX1 as well as p16^INK4a^ was observed in the plaques isolated from patients with carotid artery atherosclerosis (Jiang et al. 2022), no data so far demonstrate that CUX1‐mediated cellular senescence is involved in the pathogenesis of atherosclerosis.

In this study, we sought to identify the link between CTSL and CUX1/p16^INK4a^ in regulating atherosclerosis‐associated senescence. We discovered that CTSL can activate Notch1 proteolytically in a ligand‐independent fashion, liberating NICD. Activated NICD can transcribe CUX1 after its nuclear translocation, complexing with RBPJ. This further leads to induction of the p16^INK4a^‐dependent cellular senescence. Consistent with these findings, our immunostaining on plaques obtained from patients with carotid artery atherosclerosis demonstrates a significant induction of both CTSL and NICD in plaque zone versus normal‐appearing zone. Moreover, we further demonstrated that endothelial deletion of mouse CUX1 suppresses cellular senescence, not only in ECs (primary senescence), but also in the other types of cells throughout the entire plaques (secondary senescence), and suppression of CUX1‐mediated senescence attenuates HFD‐induced atherosclerosis in the *ApoE* ^−/−^ mice. Thus, our findings reveal that CTSL can activate Notch1 in a ligand‐independent fashion. This non‐canonical activation of Notch1 regulates CUX1/p16^INK4a^‐dependent and atherosclerosis‐associated cellular senescence, which unveils more candidate targets for senotherapeutic intervention.

## 2 Results

### 2.1 Notch1 Is a Substrate of CTSL in Human ECs

Based on our recent publications, it is known that CTSL, through its proteolytic processing of an unknown substrate, can activate *CUX1* transcription to trigger cellular senescence (Jiang et al. 2022; Wu et al. 2023, 2024). In searching for the substrate of CTSL, our Western blots cannot validate H3 and sirt1 as substrates of CTSL in human ECs, as previously reported in other cell types (Ivanov et al. 2013; Chen et al. 2012) (Figure S1). However, it was recently reported that Notch1 can be activated by cathepsin K (CTSK), a closely related cysteine protease of CTSL, in response to hypoxia in a ligand‐independent manner (Jiang et al. 2014; Yue et al. 2020), and Notch1 induces cellular senescence after being proteolytically activated to produce NICD (Kagawa et al. 2015; Rodriguez‐Vita and Fischer 2017). These findings suggest that Notch1 could be a substrate of CTSL, and NICD could induce senescence by modulating CUX1 expression.

**FIGURE 1:**
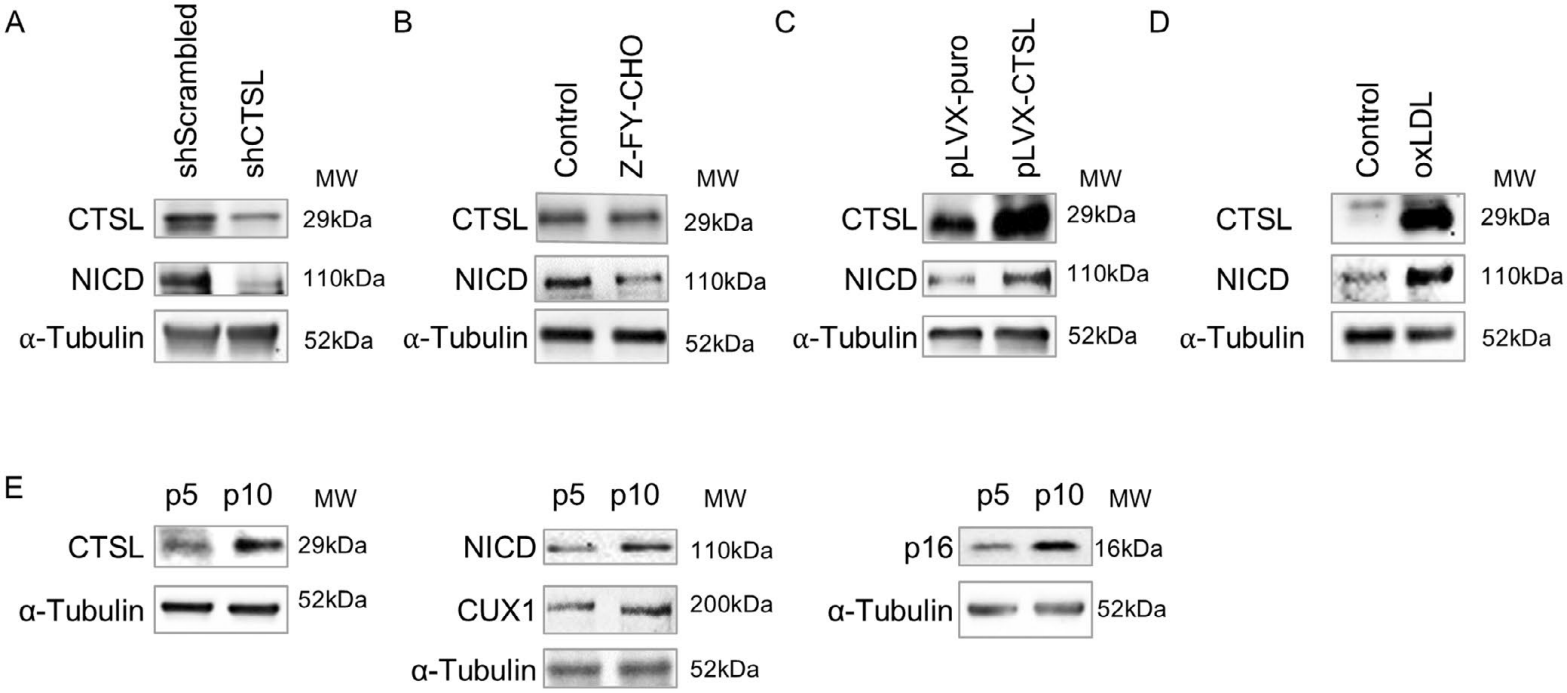
Demonstration that Notch1 is a substrate of CTSL in human ECs. (A) shRNA‐mediated CTSL knockdown (shCTSL) downregulates NICD in p10 human ECs. (B) Inhibition of CTSL by Z‐FY‐CHO downregulates NICD with CTSL protein level unchanged in p10 human ECs. (C) Overexpression of CTSL (pLVX‐CTSL) in human p5 ECs upregulates NICD. (D) Upregulation of CTSL by treating ECs with oxLDL upregulates NICD in human p5 ECs. (E) Upregulation of CTSL (Left), NICD and CUX1 (middle) and p16^INK4a^ (right) in (p10) vs. p5 human ECs. Data for Western blots represent three biologically independent samples (*n* = 3).

To demonstrate that Notch1 is a substrate of CTSL, expression of NICD was first evaluated either in the shRNA‐mediated CTSL knockdown ECs as well as in ECs with CTSL inactivated by 30 μM Z‐FY‐CHO, a potent and selective inhibitor of CTSL enzymatic activity (Kaasik et al. 2005). In both cases, a decreased level of NICD was observed (Figure 1A,B). Conversely, when CTSL was upregulated by either ectopic expression of CTSL in ECs or treating ECs with oxLDL as we recently reported (Wu et al. 2024), an induction of NICD was detected (Figure 1C,D). Moreover, recently we demonstrated that CTSL, CUX1, and p16^INK4a^ are passage‐dependent genes, and activation of these genes in late passage induces RS (Jiang et al. 2022; Wu et al. 2024). Consistently, a passage‐dependent upregulation of NICD, along with a passage‐dependent expression of CTSL, CUX1, and p16^INK4a^, was also detected (Figure 1E), which further supports that Notch1 is a substrate of CTSL. Of note, induction of cellular senescence in late passage (p10) versus early passage (p5) ECs was previously demonstrated in our lab by showing increased SA‐β‐gal and γ‐H2AX staining, SASP gene expression as well as BrdU incorporation (Jiang et al. 2022).

Together, our data demonstrate that Notch1 is a substrate of CTSL, and Notch1 can be proteolytically activated by CTSL in a passage‐dependent and ligand‐independent fashion. However, currently it is not known whether the CTSL‐mediated cleavage site on Notch 1 is different from the S3 site cleaved by γ‐secretase in a ligand‐dependent manner (Tagami et al. 2008).

### 2.2 CTSL Induces Cellular Senescence by Proteolytically Activating Notch1

**FIGURE 2:**
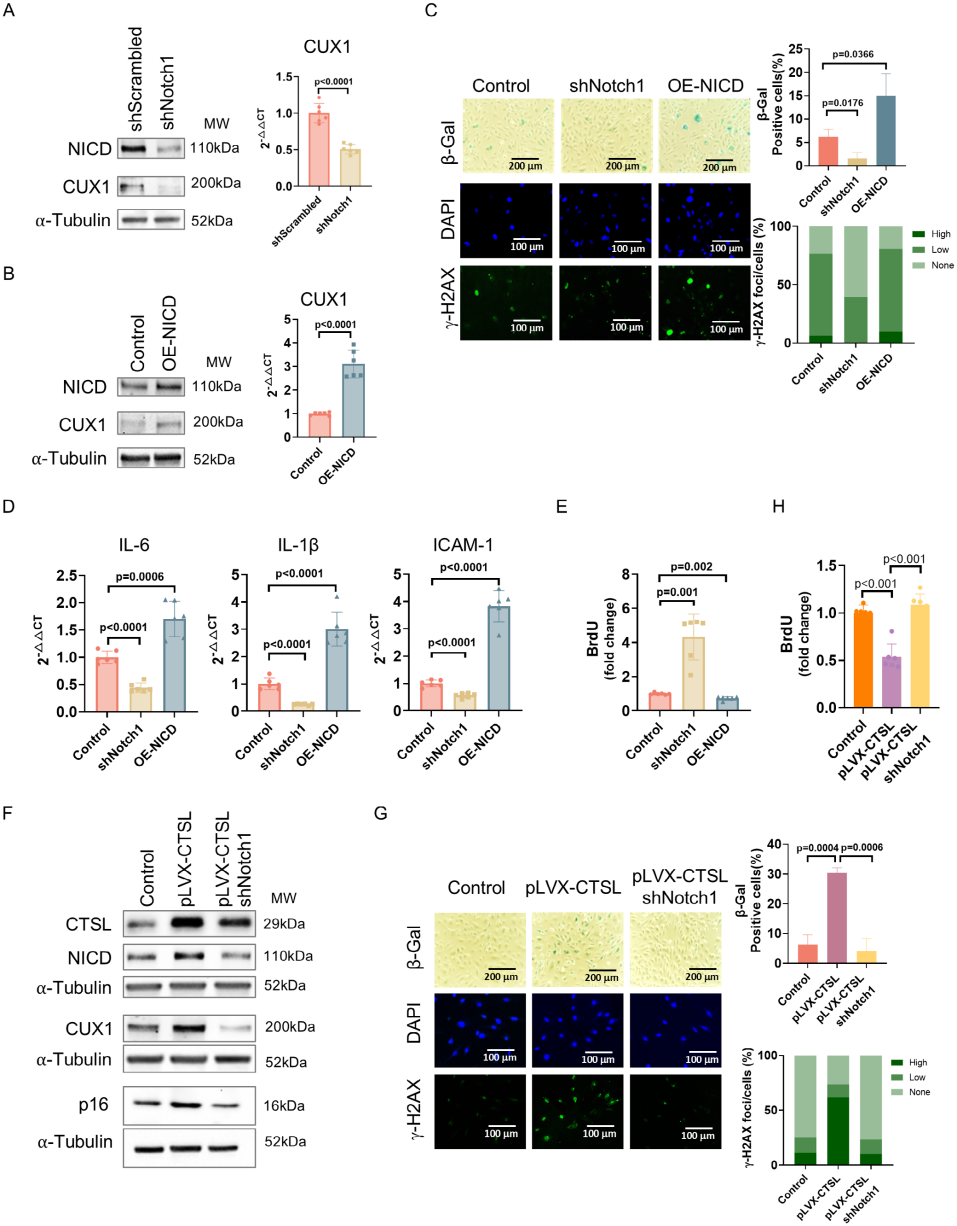
Demonstration that CTSL induces cellular senescence by proteolytically activating Notch1. (A, B) Western blots and qPCR analyses demonstrate that shRNA‐mediated Notch1 knockdown (shNotch1) in p10 ECs downregulates, whereas NICD overexpression (OE‐NICD) in p5 Ecs activates, CUX1 expression. (C–E) shRNA‐mediated Notch1 knockdown (shNotch1) in p10 Ecs suppresses, whereas NICD overexpression (OE‐NICD) in p5 ECs activates, cellular senescence as evidenced by SA‐β‐gal and γ‐H2AX staining, BrdU incorporation, and expression of SASP genes *IL‐6*, *IL‐1β* and *ICAM‐1*. (F) Western blots showing that shRNA‐mediated Notch1 knockdown (shNotch1) reverses the increased expression of NICD, CUX1 and p16^INK4a^ in the CTSL overexpressing (pLVX‐CTSL) human p5 ECs. (G) SA‐β‐gal and γ‐H2AX staining showing that shRNA‐mediated Notch1 knockdown (shNotch1) reverses the increased SA‐β‐gal and γ‐H2AX staining in the CTSL overexpressing (pLVX‐CTSL) human ECs. (H) BrdU anaylses demonstrate that shRNA‐mediated Notch1 knockdown (shNotch1) reverses the cell proliferation inhibition in the CTSL overexpressing (pLVX‐CTSL) human ECs. Data for Western blots represent three biologically independent samples (*n* = 3). Data for qPCR represent a combination of three (*n* = 3) biologically independent experiments. Data for SA‐β‐gal and γ‐H2AX staining represent three biologically independent experiments (*n* = 3). Data for BrdU represent a combination of six (*n* = 6) biologically independent experiments. Quantitative plots for both β‐gal^+^ cells (%) in SA‐β‐gal staining and γ‐H2AX foci/cells (%) with γ‐H2AX staining are shown on the right side of the panel. pLVX‐puro was used as control for lentiviral overexpression. pLKO.1 empty vector was used as control for shRNA knockdown.

To demonstrate that Notch1 is a substrate of CTSL, regulating CUX1/p16^INK4a^‐dependent cellular senescence, we first investigate if Notch1 can activate CUX1, inducing senescence in human ECs. As shown in Figure 2A,B, both our Western blots and qPCR analyses showed that shRNA‐mediated Notch1 knockdown inhibited, whereas ectopically overexpressing NICD upregulated, CUX1 expression. Consistently, cellular senescence was inhibited in the shRNA‐mediated Notch1 knockdown ECs and elevated in the NICD overexpressing ECs, respectively, as evidenced by SA‐β‐gal and γ‐H2AX staining (Figure 2C), SASP gene expression (Figure 2D), and BrdU incorporation assay (Figure 2E). These results suggest that Notch1 could regulate cellular senescence by modulating CUX1.

To demonstrate that CTSL induces CUX1/p16^INK4a^‐dependent cellular senescence by activating Notch1, a functional complementation assay was carried out to evaluate cellular senescence in the CTSL overexpressing ECs, in which Notch1 is downregulated by shRNA‐mediated knockdown. While overexpression of CTSL in ECs upregulated NICD, CUX1 and p16^INK4a^ expression (Figure 2F, left and middle lanes) and induced cellular senescence (Figure 2G, left and middle columns), shRNA‐mediated Notch1 knockdown in the CTSL overexpressing ECs reversed the expression of CUX1 and p16^INK4a^ (Figure 2F, middle and right lanes) and suppressed cellular senescence (Figure 2G, middle and right columns). Conversely, in the shRNA‐mediated CTSL knockdown ECs, overexpression of NICD restored CUX1/p16^INK4a^‐dependent cellular senescence, as shown in Figure S2. Together, these data demonstrate that CTSL can proteolytically activate Notch1, liberating NICD, which transcribes CUX1, inducing p16^INK4a^‐dependent cellular senescence.

**FIGURE 3:**
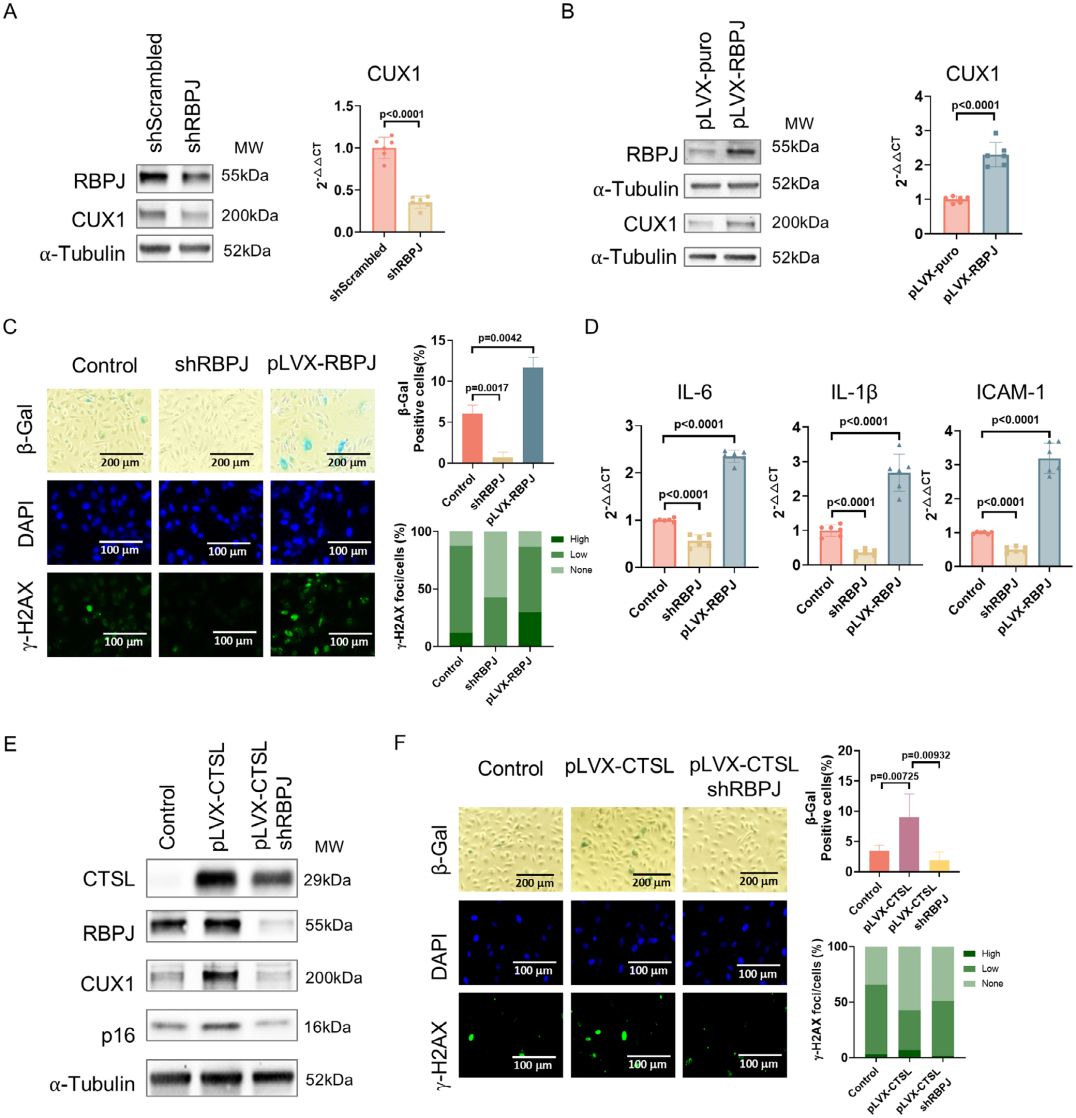
Demonstration that RBPJ is a downstream regulator of CTSL, regulating CTSL‐induced endothelial cellular senescence. (A, B) Western blots and qPCR analyses demonstrate that shRNA‐mediated RBPJ knockdown (shRBPJ) in p10 Ecs downregulates, whereas RBPJ overexpression (pLVX‐RBPJ) in p5 ECs activates, CUX1 expression. (C, D) shRNA‐mediated RBPJ knockdown (shRBPJ) in p10 ECs suppresses, whereas RBPJ overexpression (pLVX‐RBPJ) in p5 ECs activates, cellular senescence as evidenced by SA‐β‐gal and γ‐H2AX staining as well as by expression of SASP genes *IL‐6*, *IL‐1β* and *ICAM‐1*. (E) Western blots show that shRNA‐mediated RBPJ knockdown (shRBPJ) reverses the increased expression of CUX1 and p16^INK4a^ in the CTSL overexpressing (pLVX‐CTSL) human p5 ECs. (F) SA‐β‐gal and γ‐H2AX staining show that shRNA‐mediated RBPJ knockdown (shRBPJ) reverses the increased cellular senescence induced in the CTSL overexpressing (pLVX‐CTSL) human p5 ECs. Data for Western blots represent three biologically independent samples (*n* = 3). Data for qPCR represent a combination of three biologically independent experiments (*n* = 3). Data for SA‐β‐gal and γ‐H2AX staining represent three biologically independent experiments (*n* = 3). Quantitative plots for both β‐gal^+^ cells (%) in SA‐β‐gal staining and γ‐H2AX foci/cells (%) with γ‐H2AX staining are shown on the right side of the panel. pLVX‐puro was used as control for lentiviral overexpression. pLKO.1 empty vector was used as control for shRNA knockdown.

As NICD, once translocated to the nuclei, forms a transcriptional complex with RBPJ, regulating transcription of Notch1 effector genes (Kopan and Ilagan 2009; Bray 2006), this suggests that RBPJ could be a transcriptional regulator of CUX1, regulating cellular senescence. To demonstrate this, similar experiments as we performed on Notch1 shown in Figure 2 were repeated on RBPJ. As shown in Figure 3 and Figure S3, our results confirmed that RBPJ is a downstream effector of CTSL/Notch1, regulating p16^INK4a^‐dependent cellular senescence by transcriptionally activating CUX1.

Taken together, our data demonstrate that Notch1 is a substrate of CTSL, and CTSL activates Notch1/RBPJ proteolytically in a ligand‐independent fashion, inducing p16^INK4a^‐dependent cellular senescence by transcriptionally activating CUX1.

### 2.3 Inhibiting CTSL Activity and Blocking NICD Nuclear Translocation Suppress CUX1/p16^INK4a^ ‐Dependent Cellular Senescence

**FIGURE 4:**
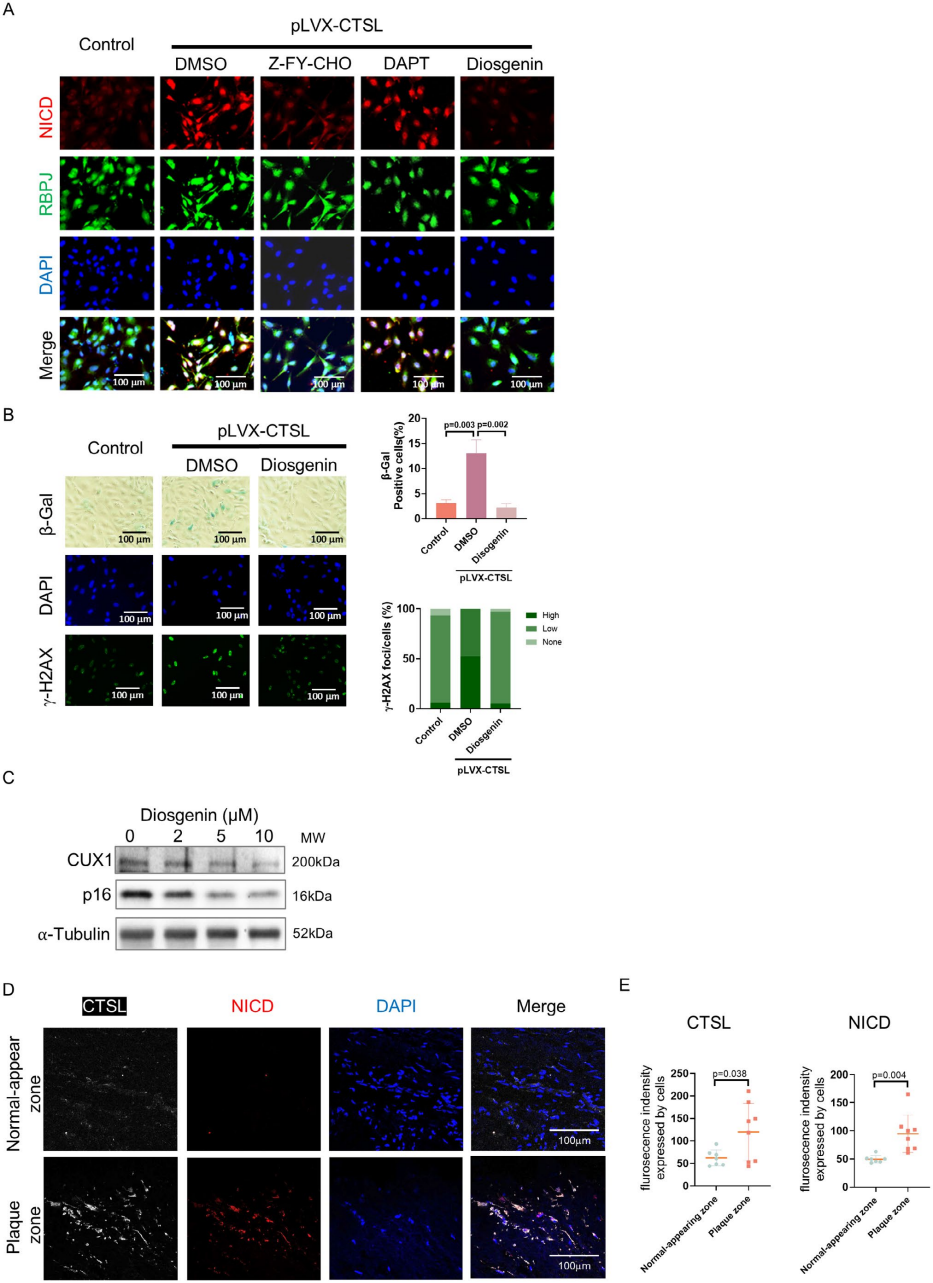
Demonstration that CTSL regulates cellular senescence via activating Notch1/RBPJ in human ECs. (A) Immunostaining show that overexpression of CTSL (pLVX‐CTSL) not only induces NICD expression, but also increases nuclear localization of NICD, which colocalized with RBPJ (Columns 1 and 2) in p5 human ECs. Treating ECs with Z‐FY‐CHO (Column 3) and diosgenin (Column 5), but not DAPT (Column 4), reduces NICD expression and blocks NICD nuclear localization and its colocalization with RBPJ. (B) SA‐β‐gal and γ‐H2AX staining show that diosgenin inhibits CTSL (pLVX‐CTSL)‐induced cellular senescence in human ECs. Data for SA‐β‐gal and γ‐H2AX staining represent three biologically independent experiments (*n* = 3). Quantitative plots for both β‐gal^+^ cells (%) in SA‐β‐gal staining and γ‐H2AX foci/cells (%) with γ‐H2AX staining are shown on the bottom of the panel. (C) Western blots showing decreased expression of CUX1 and p16^INK4a^ in p10 human ECs treated with increasing concentrations of diosgenin. Data for Western blots represent three biologically independent samples (*n* = 3). (D, E) Immunostaining with antibodies specifically against CTSL (white) and NICD (red) showing elevated expression of CTSL and NICD in the plaque zone vs. the normal‐appearing zone from patients with carotid artery atherosclerosis. Data were generated by staining of *n* = 8 plaque zone and *n* = 8 normal‐appearing zones in two independent experiments. DAPI (blue) was applied to stain fixed cells. Statistical analysis of immunostaining showing significant induction of CTSL (*p* = 0.038) and NICD (*p* = 0.004) in plaque zones compared to normal zones.

To further demonstrate that CTSL regulates CUX1/p16^INK4a^‐dependent cellular senescence by activating Notch1/RBPJ, immunostaining was performed to evaluate cellular senescence in the CTSL overexpressing human ECs. As shown in Figure 4A, overexpression of CTSL in ECs resulted in not only nuclear localization of NICD, as evidenced by colocalization of NICD with RBPJ, but also an overall increased level of NICD in the nuclei (Figure 4A, columns 1 and 2), presumably due to its stabilization after NICD complexes with RBPJ as previously reported (Liu et al. 2016; Gahr et al. 2019; Friedrich et al. 2022). Nevertheless, treating the CTSL overexpressing ECs with CTSL inhibitor Z‐FY‐CHO, but not DAPT, a well‐known inhibitor of γ‐secretase that activates canonical Notch1 signaling in a ligand dependent fashion (Geling et al. 2002), decreased NICD expression as well as its colocalization with RBPJ in the nuclei (Figure 4A, columns 3 and 4). Moreover, treating the CTSL overexpressing ECs with diosgenin, a drug that inhibits NICD nuclear translocation (Binesh et al. 2018), also decreased NICD expression and its colocalization with RBPJ in the nuclei (Figure 4A, column 5). Of note, diosgenin is a potential drug that was reported for the treatment of atherosclerosis (Binesh et al. 2018; Wang and Wang 2022). In addition, consistent with the blockage of NICD nuclear translocation, we demonstrated that, like Z‐FY‐CHO, which inhibits cellular senescence in human ECs as we recently reported (Jiang et al. 2022; Wu et al. 2024), treating human ECs with diosgenin also inhibits cellular senescence in the CTSL overexpressing human ECs, as measured by both SA‐β‐gal and γ‐H2AX staining (Figure 4B). This is accompanied by increased expression of both CUX1 and p16^INK4a^ (Figure 4C). Thus, while these results demonstrate that NICD nuclear translocation is required to induce CUX1/p16^INK4a^‐dependent cellular senescence in human ECs, our data also reveal that diosgenin prevents atherosclerosis potentially by inhibiting CUX1/p16^INK4a^‐dependent cellular senescence.

Together, our results demonstrate that CTSL regulates cellular senescence by activating the non‐canonical Notch1/RBPJ signaling, which activates CUX1, inducing p16^INK4a^‐dependent endothelial senescence.

### 2.4 Elevated CTSL and NICD Expression as Well as Cellular Senescence in the Plaques Isolated From Patients With Carotid Artery Atherosclerosis

In our recent studies, we demonstrated an upregulation of CUX1 and p16^INK4a^ in the plaques obtained from patients undergoing carotid endarterectomy. In the same plaques, we also detected an increased expression of SASP genes *IL‐6*, *IL‐1β*, and *ICAM1* (Jiang et al. 2022), suggesting that upregulation of CUX1 may promote atherosclerosis by inducing cellular senescence via modulation of *p16* ^*INK4a*^ expression. To demonstrate the role CTSL and Notch1 play in regulating CUX1/p16^INK4a^‐dependent cellular senescence in patients with atherosclerosis, we performed the same immunostaining in plaques isolated from patients with carotid artery atherosclerosis. As shown in Figure 4D,E, a significant induction of both CTSL and NICD expression was revealed in the plaque zone versus the normal‐appearing zone with *p* = 0.038 and 0.004, respectively. Consistently, a significantly elevated cellular senescence was also detected in the plaque zone versus the normal‐appearing zone as we recently described (Yan et al. 2025).

These results, together with the data previously published in our lab and by other groups showing an induction of CTSL as well as CUX1 and p16^INK4a^ in human atherosclerotic plaques (Jiang et al. 2022; Holdt et al. 2011; Liu et al. 2006), demonstrate a role that CTSL and Notch1 play in regulating CUX1/p16^INK4a^‐dependent cellular senescence in the pathogenesis of atherosclerosis.

### 2.5 Endothelial Deletion of CUX1 in Mice Attenuates Atherosclerotic Lesion and Inhibits Cellular Senescence

As aforementioned, attenuation of atherosclerotic lesion with intact and well‐organized elastin fibers and reduced macrophage content in plaque was reported in mice with CTSL inactivation (Kitamoto et al. 2007), or Notch1/RBPJ suppression (Qin et al. 2016; Binesh et al. 2018; Nus et al. 2016). However, no clear mechanism was revealed. Our findings suggest that both CTSL and Notch1 could be involved in the pathogenesis of atherosclerosis by activating CUX1/p16^INK4a^‐dependent cellular senescence. However, the role of CUX1 in atherosclerosis itself has not been addressed. To investigate this, after we confirmed the function of CUX1 in regulating p16^INK4a^‐dependent endothelial senescence in mouse aortic ECs (Figure S4), a mouse model of atherosclerosis was generated with CUX1 conditionally deleted in ECs under the *ApoE* ^*−/−*^ background (CUX1^f/f^/VE‐cadherin Cre/ApoE^−/−^, hereafter termed CUX1‐FLX). The strategy and the genotyping of the CUX1‐FLX mice were shown in Figure S5. Of note, the CUX1‐FLX mice are fertile with no obvious changes in mortality, in food and water consumption, and without anatomic abnormality of the heart or vessels as compared to the wild‐type control mice (CUX1^w/w^/VE‐cadherin Cre/ApoE^−/−^, hereafter termed CUX1‐CTRL).

**FIGURE 5:**
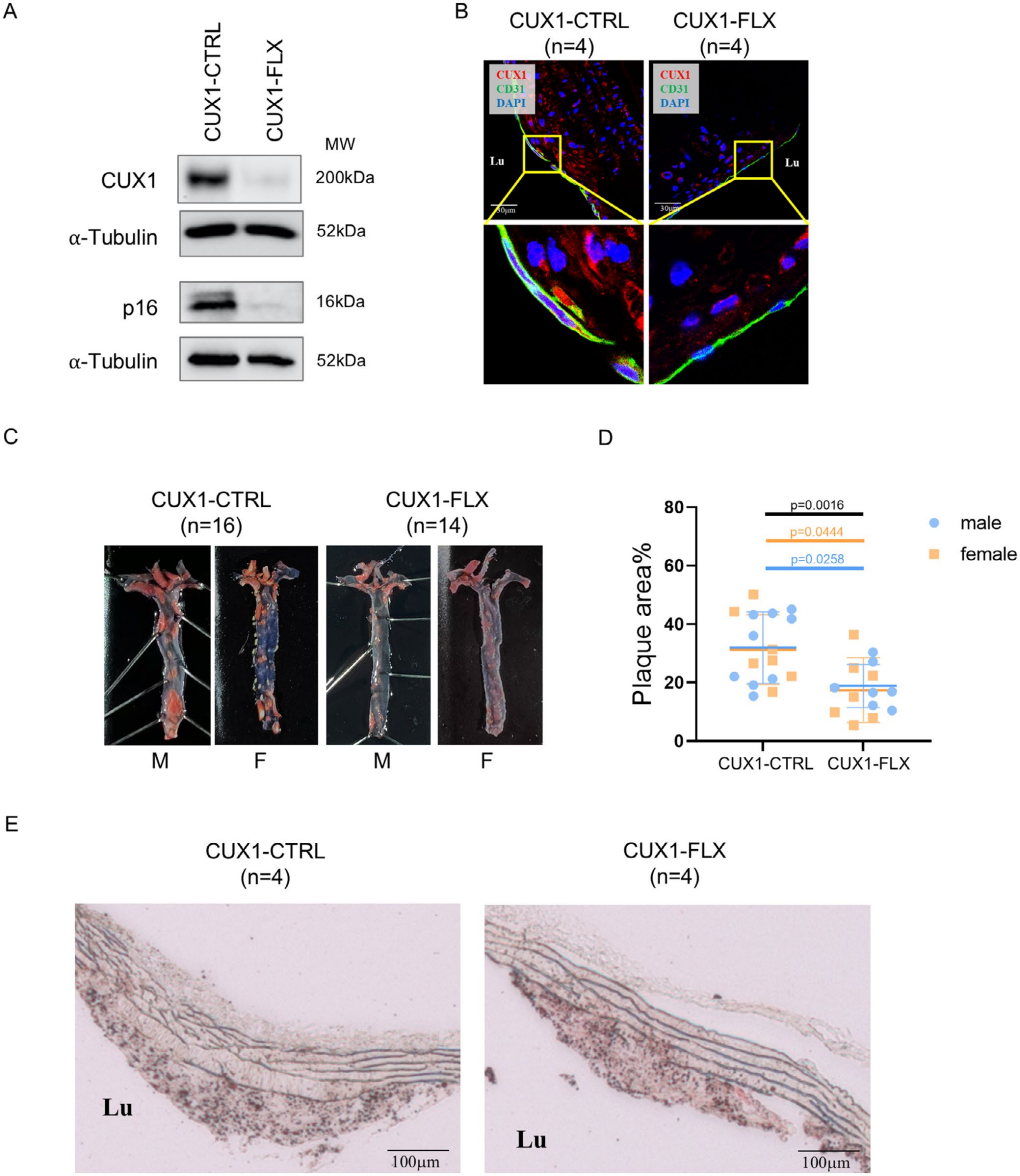
Endothelial deletion of CUX1 attenuates atherosclerotic lesion in HFD fed ApoE null mice. (A) Western blot analysis showing an inactivation of CUX1 as well as p16^INK4a^ in MLECs isolated from the CUX1‐FLX vs. the CUX1‐CTRL mice. (B) Immunostaining showing the expression of CUX1 (red) in CD31‐positive cells (green) in aortic frozen‐sections generated from the plaque zone of the CUX1‐CTRL mice (*n* = 4) (left) and the CUX1‐FLX mice (*n* = 4) (right). Sections were counterstained with DAPI (blue). (C) Representative Sudan IV enface staining of the full length aorta, including aortic arch, descending thoracic aorta and abdominal aorta, showing a decreased plaque formation in the CUX1‐FLX mice (*n* = 14; 7 males and 7 females) vs. the CUX1‐CTRL mice (*n* = 16; 9 males and 7 females) after 20 weeks of HFD feeding. (D) Quantitative plots showing a reduced percentage of plaque area in the CUX1‐FLX mice (*n* = 14; 7 males and 7 females) vs. the CUX1‐CTRL mice (*n* = 16; 9 males and 7 females). (E) ORO staining of plaques showing fragmented and disorganized elastic fibers in the lamina isolated from the CUX1‐CTRL (*n* = 4) (left) vs. the CUX1‐FLX mice (*n* = 4) (right). Data for Western blots represent three biologically independent samples (*n* = 3).

Deletion of CUX1 in ECs was confirmed by Western blots using isolated lung ECs and by immunostaining using sections of aortic roots (Figure 5A,B). Atherosclerotic lesion in the aorta, including aortic arch, descending thoracic aorta and abdominal aorta, was assessed in both CUX1‐FLX and CUX1‐CTRL mice after 20 weeks HFD feeding. As shown in Figure 5C,D, a significant reduction in atherosclerotic lesion in CUX1‐FLX versus CUX1‐CTRL mice was observed by Sudan IV enface staining of the entire aortic roots. No significant sex differences were detected (Figure 5D). In addition, we also noticed that plaques isolated from the CUX1‐FLX mice showed relatively intact and well‐organized elastic fibers in the lamina compared to the fragmented and disorganized fibers in plaques isolated from the CUX1‐CTRL (Figure 5E). As elastic fibers, composed of elastin, a crucial component of the lamina propria, providing flexibility and resilience to artery (Halabi and Kozel 2020), can be digested by elastase and elastase is a potential SASP gene (Gorgoulis et al. 2019), this result is consistent with our findings that CUX1 is involved in regulating cellular senescence. The same result is also consistent with the results obtained from the CTSL‐inactivated atherosclerosis model, showing a reduced elastin degradation in the plaque of the LDLr^−/−^/CTSL^−/−^ mice (Kitamoto et al. 2007). These data support our findings that both CTSL and CUX1 act in the same signaling pathway, inducing atherosclerosis‐associated cellular senescence.

**FIGURE 6:**
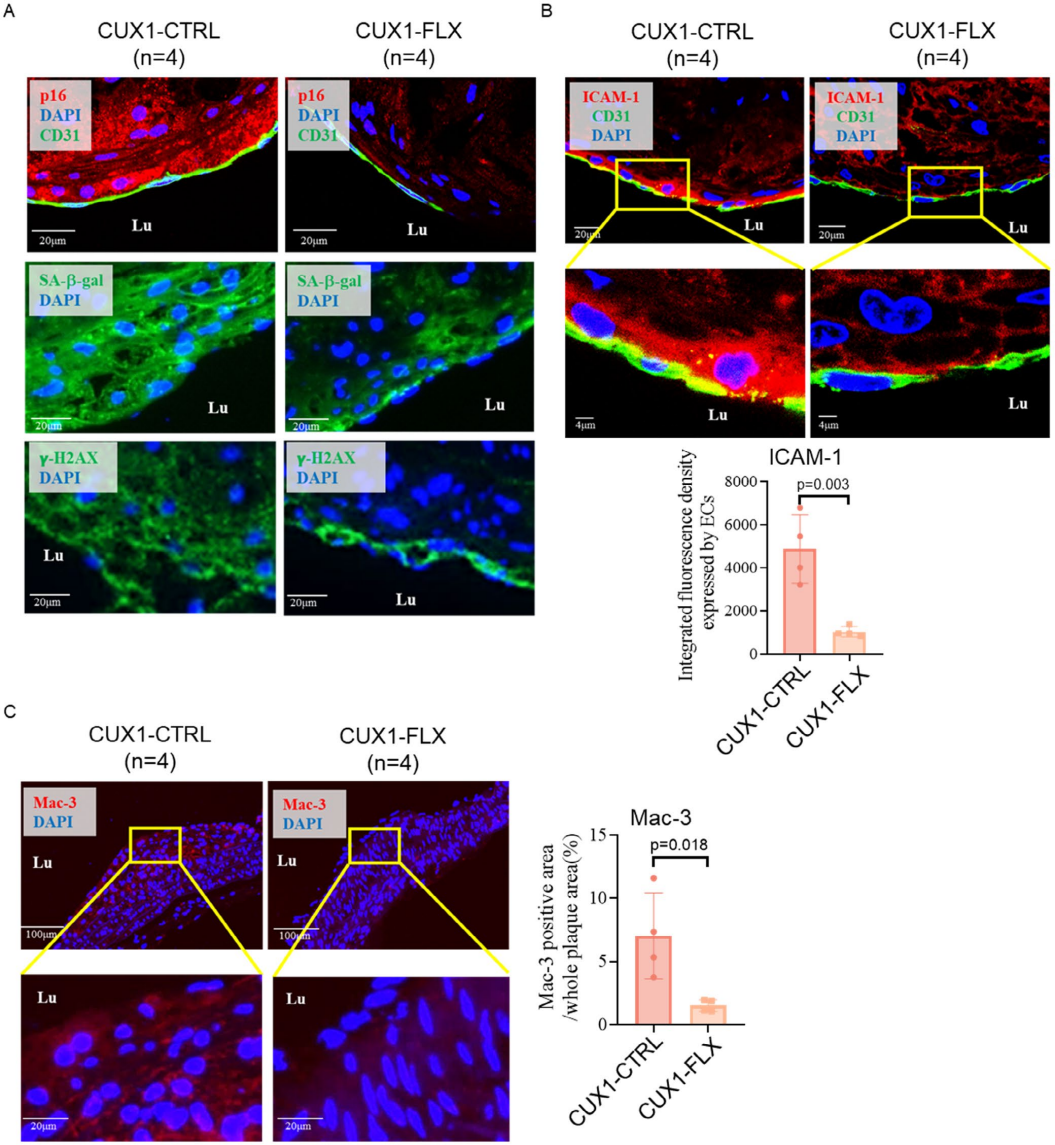
Characterization of cellular senescence in plaques isolated from the CUX1‐CTRL and CUX1‐FLX mice. (A) Immunostaining showing suppression of p16^INK4a^, SA‐β‐gal, and γ‐H2AX in plaques of the CUX1‐FLX mice (*n* = 4) (right) vs. the CUX1‐CTRL mice (*n* = 4) (right). ECs were labeled by an anti‐CD31 antibody (green). (B) Immunostaining of ICAM‐1 (red) and CD31 (green) of aortic frozen‐sections demonstrating a reduced expression of ICAM‐1 in plaques of the CUX1‐FLX mice (*n* = 4) (right) vs. the CUX1‐CTRL mice (*n* = 4) (left). Quantitative plot for ICAM‐1 staining is present on the panel below. (C) Immunostaining of macrophages using an anti‐Mac‐3 antibody (red) demonstrating a dramatical reduction of macrophages in plaques of the CUX1‐FLX mice (*n* = 4) (right) vs. the CUX1‐CTRL mice (*n* = 4) (left). Quantitative plot for Mac‐3 staining is present on the right panel. Sections were counterstained with DAPI (blue) in A–C. Student *t*‐test was used for statistical analysis.

To determine if deletion of CUX1 in ECs blocks endothelial senescence, we performed immunostaining on the plaques isolated from aortic roots using antibodies against three senescence markers including p16^INK4a^, SA‐β‐gal, and γ‐H2AX. To our surprise, while most cells in the plaques of CUX1‐CTRL mice stained positive for these three senescence markers (Figure 6A, left), very limited staining of these markers was detected in the plaques of CUX1‐FLX mice (Figure 6A, right and Figure S6). These data suggest that deletion of CUX1 in ECs is sufficient to suppress endothelial senescence (primary senescence), it also inhibits senescence in the other types of cells throughout the entire plaques (secondary senescence).

Together, our data indicate that CUX1 plays a critical role in regulating HFD‐induced senescence.

### 2.6 Endothelial Deletion of CUX1 Inhibits ICAM‐1 and Reduces Macrophage Content in Plaques

The progression of atherosclerotic plaques proceeds from the recruitment of circulating blood monocytes by ECs. The recruited monocytes enter the intima by transcytosis, where they differentiate into macrophages and become inflammatory foam cells after consuming lipid droplets (Kim et al. 2020). ICAM‐1, as a major adhesion molecule expressed in ECs, was reported to play a critical role in recruiting monocytes (Poston et al. 1992; Haydinger et al. 2023). Consistent with these previous findings, as well as our finding that ICAM‐1 is one of the SASP genes in ECs (Figures 2D and 3D and Figure S4), a high level of ICAM‐1 expression in the ECs of plaques from CUX1‐CTRL mice under HFD feeding was observed (Figure 6B, left). Consistent with the role of CUX1 in regulating cellular senescence, significantly reduced expression of ICAM‐1 was observed in CUX1‐FLX mice under the same HFD diet (Figure 6B, right). Correspondingly, as expected, more macrophages were observed in the plaques of CUX1‐CTRL mice than in CUX1‐FLX mice (Figure 6C), as measured by an anti‐Mac‐3 antibody. While these results demonstrate a role CUX1 plays in plaque progression by upregulating at least ICAM‐1 as a SASP gene, which recruits monocytes, our results are also consistent with CTSL and RBPJ models of atherosclerosis showing impaired monocyte transmigration and reduced macrophage contents in the lesion of the LDLr^−/−^/CTSL^−/−^ and ApoE^−/−^/RBPJ^−/−^ mice (Kitamoto et al. 2007; Nus et al. 2016). These data further support our finding that CTSL, Notch1/RBPJ and CUX1 act in the same signaling pathway, regulating atherosclerosis‐associated cellular senescence.

Taken together, our data suggest that CUX1‐mediated cellular senescence might not be required for plaque formation as deletion of CUX1 in ECs in CUX1‐FLX mice, in which senescence is inhibited, develops plaques as the CUX1‐CTRL mice do. Instead, CUX1‐mediated cellular senescence likely plays a key role in plaque progression as deletion of CUX1 in ECs attenuates the atherosclerotic lesion with intact and well‐organized elastic fibers and reduced plaque content of macrophages.

## 3 Discussion

Based on our recent studies showing that CTSL is an upstream activator of CUX1, inducing p16^INK4a^‐dependent cellular senescence by activating CUX1 transcription indirectly in a process that requires its proteolytic activity (Jiang et al. 2022; Wu et al. 2024), we discovered a non‐canonical Notch1 pathway as shown in graphical abstract. We demonstrated that Notch1 is a substrate of CTSL, and CTSL activates Notch1 in a ligand‐independent fashion, liberating NICD. NICD, after its nuclear translocation, complexes with RBPJ, activating CUX1/p16^INK4a^‐dependent and atherosclerosis‐associated senescence.

While our findings reveal a novel role both CTSL and Notch1/RBPJ play in the induction of cellular senescence, they raise many questions. For example, (1) how can CTSL, as a protease mainly stored in lysosomes, cleave Notch1 on the plasma membrane in the cytosol? (2) Does ligand‐dependent/γ‐secretase‐mediated Notch1 activation also induce cellular senescence? If not, how will the ligand‐dependent/γ‐secretase‐mediated Notch1 activation differentiate from the ligand‐independent/CTSL‐mediated Notch1 activation? (3) Are other Notch family genes Notch2, 3, and 4 substrates of CTSL? (4) Can other cathepsins activate Notch1 to induce cellular senescence? Once these questions as well as many other questions are answered, we will have a better understanding about how p16^INK4a^‐dependent cellular senescence is activated by Notch1 signaling, which, we believe, will provide new insights into the development of drugs for preventing atherosclerosis and other age‐related diseases.

In addition, consistent with prior observations that show attenuation of atherosclerotic lesion with reduced plaque macrophage contents in the mouse models of atherosclerosis with CTSL inactivation (Kitamoto et al. 2007) and Notch1/RBPJ suppression (Qin et al. 2016; Binesh et al. 2018; Nus et al. 2016), we observed the same phenotypes in the CUX1‐FLX mice. These data further support our findings that all these proteins are involved in regulating atherosclerosis‐associated cellular senescence in the same signaling pathway.

Finally, based on our observation that deletion of CUX1 in ECs can block cellular senescence throughout the entire plaques, we think that HFD feeding can directly induce primary senescence in ECs. While senescent ECs can recruit monocytes to the lesion sites by overexpressing adhesion molecules, including ICAM‐1 as part of the SASP, senescent ECs can induce secondary senescence in other types of cells such as VSMCs and foamy macrophages throughout the entire plaques. This explains why deletion of CUX1 in ECs can inhibit not only endothelial senescence (primary senescence) but also senescence in other types of cells within plaques (secondary senescence). Of note, secondary senescence is generally induced by SASP through either juxtacrine and/or paracrine signaling (Childs et al. 2018). Even though we have detected the induction of SASP gene expression by qPCR as one of the markers for senescence in vitro, our data only support senescence‐associated transcriptional upregulation of these SASP genes during CTSL‐induced and Notch1‐mediated senescence. To determine if juxtacrine or paracrine signaling is involved in HFD‐induced secondary senescence in vivo, detection of the secreted form of SASP factors in mice is warranted even though it is technically difficult to measure the local secretion of SASP factors. In addition, our observation also raises a question about how endothelial senescence is activated by HFD. Is it activated from the side of the arterial lumen or from the side of the intima within a plaque? Different answers to this question could lead to different strategies to prevent plaque progression by inhibiting HFD‐induced primary senescence in ECs.

## 4 Materials and Methods

### 4.1 Cell Culture and Reagents

HUVECs (Cat#: PCS‐100‐013) were purchased from Lonza and cultured in human EC growth medium V2 (Lonza, Cat#: CC‐3183). Primary mouse arterial ECs (MAECs) were purchased from Cell Biologics (Cat#: C57‐6052) and were cultured in complete endothelial cell medium (Cell Biologics, Cat#: M1168). All cells were cultured at 37°C in 5% CO_2_.

### 4.2 Chemicals

HUVECs were treated with 30 μM Z‐FY‐CHO (Santa Cruz, Cat#: sc‐3132) for 48 h; 0.1 mg/mL oxLDL for 48 h; 5 μM Diosgenin (Cayman, Cat#: 512‐04‐9) for 48 h; and 10 μM DAPT (Abcam, Cat#: 120633) for 48 h.

### 4.3 Generation of CUX1^f^ ^/f^ Mice

All animal studies were carried out in accordance with the protocols approved by the Institutional Animal Care and Use Committee of the University of Pittsburgh (#23063387). CUX1^f/f^ mice were generated in C57BL/6J mice using CRISPR‐Cas9 technology as previously described (Pelletier et al. 2015). The strategy and the genotyping of the CUX1^f/f^ mice were presented in Figure S5. sgRNA CGACCGTCGAGTTTAGGTGCTGG and CCGTCCCCGAGAGTGAGGCGTGG, as well as the repair guide DNA were used. No off‐target sites were identified using the Cas‐OFFinder algorithm (Bae et al. 2014). Two independent founders were generated to establish two CUX1^f/f^‐mouse lines that were used for functional analysis. Both the VE‐cadherin Cre mice and the ApoE^−/−^ mice were purchased from the Jackson lab and genotyped according to Jackson lab's instruction.

### 4.4 Mouse Lung Endothelial Cells (MLECs) Isolation

MLECs were isolated following the protocol for lung endothelial cell isolation (Reynolds and Hodivala‐Dilke 2006). Briefly, mouse lungs were digested with type I collagenase and plated on gelatin and collagen‐coated flasks. The cells were then subjected to sequential negative sorting by magnetic beads coated with a sheep anti‐rat antibody using an Fc Blocker (BD Pharmingen; rat anti‐mouse CD16/CD32, Cat#: 553142) to remove macrophages and for positive‐sorting by magnetic beads using an anti‐ICAM2 (or CD102) antibody (BD Pharmingen, Cat#: 553326) to isolate endothelial cells. MLECs were cultured in DMEM plus 20% FBS, 1X endothelial cellgrowth supplement (ECGS) and 100ug/ml heparin.

### 4.5 Atherosclerotic Lesion Characterization

For characterization of plaque formation, the CUX1‐FLX mice and the CUX1‐CTRL mice were euthanized by CO_2_ asphyxiation. Mouse aortas were dissected in cold PBS and cut open to expose the atherosclerotic plaques. After fixation in 4% formaldehyde at 4°C for 24 h, tissues were first rinsed in PBS for 10 min and then in 70% EtOH for 5 min. The dissected aortas were then stained with Sudan IV for 7 min with gentle shaking. After rinsing in 80% EtOH and PBS, images were taken using a SONY RX100VI Camera. The plaque areas were analyzed using National Institutes of Health ImageJ 1.52a software and calculated by expressing the plaque area relative to the total vascular area.

### 4.6 Histological Analysis

For histological staining, cryostat sections (6 μm) of the aortic arch were cut and used as previously described (Kitamoto et al. 2007). For Oil Red O (ORO) staining, sections were first rinsed with H_2_O, dehydrated with 60% isopropanol, and then stained with freshly prepared ORO solutions for 30 min in the dark. After ORO staining, sections were quickly rinsed with 60% isopropanol and washed with ddH_2_O until the solution was clear. Sections were then counterstained with 10% Mayer's hematoxylin, photographed under laser microscopy, and analyzed with National Institutes of Health ImageJ 1.52a software.

### 4.7 Primers and Antibodies

All primers used in this study were purchased from IDT and are listed in Table S1. All antibodies used are listed in Table S2 with the corresponding supplier information.

### 4.8 Isolation of Atherosclerotic Plaques From Patients With Carotid Artery Atherosclerosis

Atherosclerotic plaques were obtained from patients undergoing carotid endarterectomy at the Department of Surgery at UPMC. For the use of human materials, written informed consent was obtained from all individuals before operative procedures, as approved by the University of Pittsburgh (Institutional Review Board no. STUDY18100138).

### 4.9 qPCR Analysis

Total RNA was isolated using the RNeasy Mini kit (Qiagen, Cat#: 74104). cDNA was synthesized using SuperScript III Reverse Transcriptase (ThermoFisher, Cat#: 10880093) after the RNA samples were treated with DNase I (ThermoFisher, Cat#: EN0525). All the procedures were performed following the manufacturer's protocols. qPCR was performed with the StepOne real‐time PCR system according to the protocol for the Power SYBR Green PCR Master Mix (Applied Biosystems) and for TaqMan Universal PCR Master Mix (ThermoFisher, Cat#: 4364340). The following probe/primer mixes for TaqMan PCR were purchased from Applied Biosystems: p16 Hs02902543_mH and GAPDH internal control (Hs02786624_g1). Other primers used are listed in Table S1. The data represent the combination of three independent samples (*n* = 3).

### 4.10 Western Blot Analysis

Whole cell lysates prepared using RIPA buffer (Sigma, Cat#: 89900) were used for Western blot analysis. Proteins were resolved on SDS‐PAGE gels, transferred to PVDF membranes, and then detected using gene‐specific antibodies. All antibodies were purchased and used as listed in Table S2. For a loading control, α‐tubulin was used. The data represent three independent biological replicates (*n* = 3). The data obtained from the Western blots are quantified by densitometry analysis as presented in Figure S7.

### 4.11 BrdU Proliferation Assay

Human ECs proliferation was determined by BrdU incorporation using the BrdU Cell Proliferation Assay Kit (Cell Signaling, Cat#: 6813). Briefly, human ECs were sub‐cultured (10,000 cells per well) in 96‐well plates for 24 h after virus injection. Then, 1× BrdU was added to the culture medium for DNA labeling. The labeling medium was removed after 2 h and, cells were fixed, and DNA was denatured by the addition of 100 μL fixing/denaturing solution for 30 min. The incorporated BrdU was then detected by a mouse anti‐BrdU monoclonal antibody and measured using an anti‐mouse IgG, horseradish peroxidase‐linked antibody following the manufacturers' instructions. Data for the BrdU proliferation assay represent *n* = 6 independent biological samples.

### 4.12 Senescence‐Associated β‐Galactosidase Staining in Cells

SA‐β‐Gal Staining Kit (Cell Signaling, Cat#: 9860S) was used to stain senescent HUVECs. In brief, cells were evenly distributed on the cell culture plate. After preliminary treatment, cells were fixed with the provided fixative for 15 min, then washed, followed by staining with β‐Gal staining reagent, and incubated at 37 degrees for 24 h. The number of β‐Gal positive cells is then counted. Visualization was done using an RVL‐100‐G microscope (Echo Laboratories, San Diego, CA, USA). Images were analyzed using ImageJ software (version 1.52 K, NIH). The data represent three independent biological replicates (*n* = 3).

### 4.13 Immunofluorescence Staining in Cells

Cells were plated on glass coverslips and fixed in 4% paraformaldehyde. For staining, cells were blocked in PBS containing 5% FBS and 0.5% Triton X‐100 for 1 h at room temperature. Cells were first incubated with antibodies (listed in Table S2) in the blocking buffer overnight at 4°C and immunofluorescence was detected with Alexa Fluor 488/594‐conjugated secondary antibody for 1 h at room temperature. Cells were counterstained with 4′, 6‐diamidino‐2‐phenylindole (DAPI) (Sigma, Cat# D9542). Visualization was carried out using an RVL‐100‐G microscope (Echo Laboratories, San Diego, CA, USA). Images were analyzed using ImageJ software (version 1.52 K, NIH). The data represent three independent biological replicates (*n* = 3).

### 4.14 Immunofluorescence Staining of SA‐β‐Gal, γ‐H2AX, p16^INK4a^ , MAC‐3, CTSL, NICD and ICAM‐1 in Tissue Cryosections

Human atherosclerotic plaques were obtained from patients undergoing carotid endarterectomy. Part of the carotid artery that shows calcified hard tissue was used as a plaque zone and part that is far from the calcified zone was used as a normal‐appearing zone. The data represent two independent experiments with eight plaque zones (*n* = 8) and eight normal‐appearing zones (*n* = 8). Both plaque and normal‐appearing zones were separated and fixed with 4% buffered formalin for 2 h and stored in 30% sucrose solution containing 0.05% sodium azide overnight. Sections were made of 10 μm thickness, permeabilized with 0.1% triton X‐100 for 4 h, and blocked overnight in PBS containing 2% BSA. For staining with mice cryostat sections, cryostat sections (6 μm) were permeabilized with 0.1% triton X‐100 for 4 h and blocked in PBS containing 2% BSA overnight. Sections were further incubated for 24 h with primary antibodies against SA‐β‐gal, γ‐H2AX, p16^INK4a^, MAC‐3, CTSL, NICD, or ICAM‐1. After washing with PBS, sections were incubated for 1 h at room temperature with fluorochrome conjugated secondary antibodies. Tissue sections were stained and mounted with VECTASHIELD DAPI. Images were taken using confocal laser microscopy and analyzed using ImageJ.

### 4.15 RNAi Knockdown

For siRNA transient knockdown in mouse aorta ECs, siRNAs for mouse CUX1 were purchased from Thermo Fisher (Cat#: AM16708) and transfected using Lipofectamin RNAi MAX reagent (Thermo Fisher, Cat#: 13778075). Knockdown of CUX1 was detected by qPCR using the Power SYBR Green PCR Master Mix (Applied Biosystems) (Thermo Fisher, Cat#: 4367659) according to the manufacturer's protocol.

For shRNA‐mediated CTSL and RBPJ knockdown in HUVECs, lentiviruses were generated (targeted sequences are listed in Table S1) using the pLKO.1‐puro vector (Addgene, Plasmid #8453) by transfecting 293 T cells and used to infect HUVECs. shRNA‐mediated Notch1 knockdown virus (Cat#: TRCN0000003359) was bought from Sigma. Assays for cellular senescence, including SA‐β‐Gal and γ‐H2AX staining as well as SASP gene expression, were performed 48 h after shRNA lentiviral infection of HUVECs.

### 4.16 Overexpression of CTSL, RBPJ, and NICD

For overexpression of human CTSL, RBPJ, and NICD, human CTSL, RBPJ, and NICD cDNAs were cloned into the pLVX‐puro vector (Clontech, Cat#: 632164). After being confirmed by sequencing, they were overexpressed using a lentiviral expression vector. Lentiviruses were generated by transfecting 293 T cells and were used to infect HUVECs.

### 4.17 Statistical Analysis

All data were presented as mean ± standard deviation (SD) and analyzed using the Prism 8 software (GraphPad) package. The qPCR results were normalized, with the control group set to an average value of 1 after normalization. The experimental group was then assigned values based on this and subjected to statistical analysis. When comparing two groups, a two‐tailed Student's *t*‐test was applied after testing for normal distribution. Non‐normally distributed data relating to quantification of immunocytochemical staining in Figure 4D are presented as median ± interquartile range, and *p* values were calculated with the nonparametric Mann–Whitney test for pairwise comparison. When designing comparisons among multiple experimental groups, the statistical significance of the differences between groups was assessed using two‐tailed Student's *t*‐test for pairwise comparisons or one‐way analysis of variance (ANOVA) followed by a post hoc Student–Newman–Keuls test for multiple comparisons. For all tests, *p* values < 0.05 were considered statistically significant.

All data and reagents are available upon reasonable request.

## Author Contributions

G.L. designed the study, analyzed the data, and drafted and revised the manuscript; Y.W. performed all the experiments and participated in drafting the manuscript; D.J., X.T., and T.W. performed the work on regulation of CUX1 by CTSL. J.L., Z.D. and Q.W. assisted mice work. L.L., and S.Y. assisted with Western blot and qPCR analyses; J.E., and P.D. performed immunostaining using atherosclerotic plaques. W.S. assisted with the work on VSMCs; J.K.A., J.X.T., and P.D. assisted with data analysis and manuscript revision.

## Funding

This work was supported partly by grants from NIH NIA R01AG056279 (GL), R01AG065229 (GL), and PA Department of Health SAP#4100087331 (GL).

## Conflicts of Interest

The authors declare no conflicts of interest.

## Supporting information


**Appendix S1:** acel70563‐sup‐0001‐AppendixS1.docx.

## Data Availability

The data that support the findings of this study are available on request from the corresponding author. The data are not publicly available due to privacy or ethical restrictions.

## References

[acel70563-bib-0001] Admasu, T. D. , M. Rae , and A. Stolzing . 2021. “Dissecting Primary and Secondary Senescence to Enable New Senotherapeutic Strategies.” Ageing Research Reviews 70: 101412. 10.1016/j.arr.2021.101412. 34302996

[acel70563-bib-0002] Bae, S. , J. Park , and J. S. Kim . 2014. “Cas‐OFFinder: A Fast and Versatile Algorithm That Searches for Potential Off‐Target Sites of Cas9 RNA‐Guided Endonucleases.” Bioinformatics 30: 1473–1475. 10.1093/bioinformatics/btu048. 24463181 PMC4016707

[acel70563-bib-0003] Baker, D. J. , B. G. Childs , M. Durik , et al. 2016. “Naturally Occurring p16 Ink4a‐Positive Cells Shorten Healthy Lifespan.” Nature 530: 184–189. 26840489 10.1038/nature16932 PMC4845101

[acel70563-bib-0004] Baker, D. J. , T. Wijshake , T. Tchkonia , et al. 2011. “Clearance of p16Ink4a‐Positive Senescent Cells Delays Ageing‐Associated Disorders.” Nature 479: 232–236. 10.1038/nature10600. 22048312 PMC3468323

[acel70563-bib-0005] Binesh, A. , S. N. Devaraj , and H. Devaraj . 2018. “Inhibition of Nuclear Translocation of Notch Intracellular Domain (NICD) by Diosgenin Prevented Atherosclerotic Disease Progression.” Biochimie 148: 63–71. 10.1016/j.biochi.2018.02.011. 29481959

[acel70563-bib-0006] Bray, S. J. 2006. “Notch Signalling: A Simple Pathway Becomes Complex.” Nature Reviews Molecular Cell Biology 7: 678–689. 10.1038/nrm2009. 16921404

[acel70563-bib-0007] Cai, Z. , B. Zhao , Y. Deng , et al. 2016. “Notch Signaling in Cerebrovascular Diseases (Review).” Molecular Medicine Reports 14: 2883–2898. 10.3892/mmr.2016.5641. 27574001 PMC5042775

[acel70563-bib-0008] Chen, J. , S. Xavier , E. Moskowitz‐Kassai , et al. 2012. “Cathepsin Cleavage of Sirtuin 1 in Endothelial Progenitor Cells Mediates Stress‐Induced Premature Senescence.” American Journal of Pathology 180: 973–983. 10.1016/j.ajpath.2011.11.033. 22234173 PMC3349894

[acel70563-bib-0009] Childs, B. G. , H. Li , and J. M. van Deursen . 2018. “Senescent Cells: A Therapeutic Target for Cardiovascular Disease.” Journal of Clinical Investigation 128: 1217–1228. 10.1172/JCI95146. 29608141 PMC5873883

[acel70563-bib-0010] Dang, Y. , Y. An , J. He , et al. 2020. “Berberine Ameliorates Cellular Senescence and Extends the Lifespan of Mice via Regulating p16 and Cyclin Protein Expression.” Aging Cell 19: e13060. 10.1111/acel.13060. 31773901 PMC6974710

[acel70563-bib-0011] Friedrich, T. , F. Ferrante , L. Pioger , et al. 2022. “Notch‐Dependent and ‐Independent Functions of Transcription Factor RBPJ.” Nucleic Acids Research 50: 7925–7937. 10.1093/nar/gkac601. 35848919 PMC9371899

[acel70563-bib-0012] Gahr, B. M. , F. Brandle , M. Zimmermann , and A. C. Nagel . 2019. “An RBPJ‐Drosophila Model Reveals Dependence of RBPJ Protein Stability on the Formation of Transcription‐Regulator Complexes.” Cells 8: 1252. 10.3390/cells8101252. 31615108 PMC6829621

[acel70563-bib-0013] Geling, A. , H. Steiner , M. Willem , L. Bally‐Cuif , and C. Haass . 2002. “A Gamma‐Secretase Inhibitor Blocks Notch Signaling In Vivo and Causes a Severe Neurogenic Phenotype in Zebrafish.” EMBO Reports 3: 688–694. 10.1093/embo-reports/kvf124. 12101103 PMC1084181

[acel70563-bib-0014] Gorgoulis, V. , P. D. Adams , A. Alimonti , et al. 2019. “Cellular Senescence: Defining a Path Forward.” Cell 179: 813–827. 10.1016/j.cell.2019.10.005. 31675495

[acel70563-bib-0015] Halabi, C. M. , and B. A. Kozel . 2020. “Vascular Elastic Fiber Heterogeneity in Health and Disease.” Current Opinion in Hematology 27: 190–196. 10.1097/MOH.0000000000000578. 32141894 PMC7325869

[acel70563-bib-0016] Haydinger, C. D. , L. M. Ashander , A. C. R. Tan , and J. R. Smith . 2023. “Intercellular Adhesion Molecule 1: More Than a Leukocyte Adhesion Molecule.” Biology‐Basel 12: 743. 10.3390/biology12050743. 37237555 PMC10215618

[acel70563-bib-0017] Head, T. , S. Daunert , and P. J. Goldschmidt‐Clermont . 2017. “The Aging Risk and Atherosclerosis: A Fresh Look at Arterial Homeostasis.” Frontiers in Genetics 8: 216. 10.3389/fgene.2017.00216. 29312440 PMC5735066

[acel70563-bib-0018] Helgadottir, A. , G. Thorleifsson , A. Manolescu , et al. 2007. “A Common Variant on Chromosome 9p21 Affects the Risk of Myocardial Infarction.” Science 316: 1491–1493. 10.1126/science.1142842. 17478679

[acel70563-bib-0019] Hickson, L. J. , L. G. P. Langhi Prata , S. A. Bobart , et al. 2019. “Senolytics Decrease Senescent Cells in Humans: Preliminary Report From a Clinical Trial of Dasatinib Plus Quercetin in Individuals With Diabetic Kidney Disease.” eBioMedicine 47: 446–456. 10.1016/j.ebiom.2019.08.069. 31542391 PMC6796530

[acel70563-bib-0020] Hoare, M. , Y. Ito , T. W. Kang , et al. 2016. “NOTCH1 Mediates a Switch Between Two Distinct Secretomes During Senescence.” Nature Cell Biology 18: 979–992. 10.1038/ncb3397. 27525720 PMC5008465

[acel70563-bib-0021] Holdt, L. M. , K. Sass , G. Gabel , H. Bergert , J. Thiery , and D. Teupser . 2011. “Expression of Chr9p21 Genes CDKN2B (p15(INK4b)), CDKN2A (p16(INK4a), p14(ARF)) and MTAP in Human Atherosclerotic Plaque.” Atherosclerosis 214: 264–270. 10.1016/j.atherosclerosis.2010.06.029. 20637465

[acel70563-bib-0022] Ivanov, A. , J. Pawlikowski , I. Manoharan , et al. 2013. “Lysosome‐Mediated Processing of Chromatin in Senescence.” Journal of Cell Biology 202: 129–143. 10.1083/jcb.201212110. 23816621 PMC3704985

[acel70563-bib-0023] Jiang, D. , W. Sun , T. Wu , et al. 2022. “Post‐GWAS Functional Analysis Identifies CUX1 as a Regulator of p16INK4a and Cellular Senescence.” Nature Aging 2: 140–154. 10.1038/s43587-022-00177-0. 37117763 PMC10154215

[acel70563-bib-0024] Jiang, H. , X. W. Cheng , G. P. Shi , et al. 2014. “Cathepsin K‐Mediated Notch1 Activation Contributes to Neovascularization in Response to Hypoxia.” Nature Communications 5: 3838. 10.1038/ncomms4838. 24894568

[acel70563-bib-0025] Kaasik, A. , T. Rikk , A. Piirsoo , T. Zharkovsky , and A. Zharkovsky . 2005. “Up‐Regulation of Lysosomal Cathepsin L and Autophagy During Neuronal Death Induced by Reduced Serum and Potassium.” European Journal of Neuroscience 22: 1023–1031. 10.1111/j.1460-9568.2005.04279.x. 16176344

[acel70563-bib-0026] Kagawa, S. , M. Natsuizaka , K. A. Whelan , et al. 2015. “Cellular Senescence Checkpoint Function Determines Differential Notch1‐Dependent Oncogenic and Tumor‐Suppressor Activities.” Oncogene 34: 2347–2359. 10.1038/onc.2014.169. 24931169 PMC4268095

[acel70563-bib-0027] Khosla, S. , J. N. Farr , T. Tchkonia , and J. L. Kirkland . 2020. “The Role of Cellular Senescence in Ageing and Endocrine Disease.” Nature Reviews Endocrinology 16: 263–275. 10.1038/s41574-020-0335-y. PMC7227781 32161396

[acel70563-bib-0028] Kim, K. W. , S. Ivanov , and J. W. Williams . 2020. “Monocyte Recruitment, Specification, and Function in Atherosclerosis.” Cells 10: 15. 10.3390/cells10010015. 33374145 PMC7823291

[acel70563-bib-0029] Kitamoto, S. , G. K. Sukhova , J. Sun , et al. 2007. “Cathepsin L Deficiency Reduces Diet‐Induced Atherosclerosis in Low‐Density Lipoprotein Receptor‐Knockout Mice.” Circulation 115: 2065–2075. 10.1161/CIRCULATIONAHA.107.688523. 17404153

[acel70563-bib-0030] Kopan, R. , and M. X. Ilagan . 2009. “The Canonical Notch Signaling Pathway: Unfolding the Activation Mechanism.” Cell 137: 216–233. 10.1016/j.cell.2009.03.045. 19379690 PMC2827930

[acel70563-bib-0031] Liu, J. , J. X. Shen , X. F. Wen , Y. X. Guo , and G. J. Zhang . 2016. “Targeting Notch Degradation System Provides Promise for Breast Cancer Therapeutics.” Critical Reviews in Oncology/Hematology 104: 21–29. 10.1016/j.critrevonc.2016.05.010. 27263934

[acel70563-bib-0032] Liu, J. , G. K. Sukhova , J. T. Yang , et al. 2006. “Cathepsin L Expression and Regulation in Human Abdominal Aortic Aneurysm, Atherosclerosis, and Vascular Cells.” Atherosclerosis 184: 302–311. 10.1016/j.atherosclerosis.2005.05.012. 15982660

[acel70563-bib-0033] Liu, Z. J. , Y. Tan , G. W. Beecham , et al. 2012. “Notch Activation Induces Endothelial Cell Senescence and Pro‐Inflammatory Response: Implication of Notch Signaling in Atherosclerosis.” Atherosclerosis 225: 296–303. 10.1016/j.atherosclerosis.2012.04.010. 23078884 PMC3502717

[acel70563-bib-0034] McPherson, R. , A. Pertsemlidis , N. Kavaslar , et al. 2007. “A Common Allele on Chromosome 9 Associated With Coronary Heart Disease.” Science 316: 1488–1491. 10.1126/science.1142447. 17478681 PMC2711874

[acel70563-bib-0035] Mijit, M. , V. Caracciolo , A. Melillo , F. Amicarelli , and A. Giordano . 2020. “Role of p53 in the Regulation of Cellular Senescence.” Biomolecules 10: 420. 10.3390/biom10030420. 32182711 PMC7175209

[acel70563-bib-0036] Nus, M. , B. Martinez‐Poveda , D. MacGrogan , et al. 2016. “Endothelial Jag1‐RBPJ Signalling Promotes Inflammatory Leucocyte Recruitment and Atherosclerosis.” Cardiovascular Research 112: 568–580. 10.1093/cvr/cvw193. 27496872

[acel70563-bib-0037] Palmer, W. H. , and W. M. Deng . 2015. “Ligand‐Independent Mechanisms of Notch Activity.” Trends in Cell Biology 25: 697–707. 10.1016/j.tcb.2015.07.010. 26437585 PMC4628868

[acel70563-bib-0038] Pelletier, S. , S. Gingras , and D. R. Green . 2015. “Mouse Genome Engineering via CRISPR‐Cas9 for Study of Immune Function.” Immunity 42: 18–27. 10.1016/j.immuni.2015.01.004. 25607456 PMC4720985

[acel70563-bib-0039] Poston, R. N. , D. O. Haskard , J. R. Coucher , N. P. Gall , and R. R. Johnson‐Tidey . 1992. “Expression of Intercellular Adhesion Molecule‐1 in Atherosclerotic Plaques.” American Journal of Pathology 140: 665–673. 1372160 PMC1886152

[acel70563-bib-0040] Qin, W. D. , F. Zhang , X. J. Qin , et al. 2016. “Notch1 Inhibition Reduces Low Shear Stress‐Induced Plaque Formation.” International Journal of Biochemistry & Cell Biology 72: 63–72. 10.1016/j.biocel.2016.01.007. 26783939

[acel70563-bib-0041] Reynolds, L. E. , and K. M. Hodivala‐Dilke . 2006. “Primary Mouse Endothelial Cell Culture for Assays of Angiogenesis.” Methods in Molecular Medicine 120: 503–509. 10.1385/1-59259-969-9:503. 16491622

[acel70563-bib-0042] Rodriguez‐Vita, J. , and A. Fischer . 2017. “Notch1 Induces Endothelial Senescence and Promotes Tumor Progression.” Cell Cycle 16: 911–912. 10.1080/15384101.2017.1316575. 28426366 PMC5462074

[acel70563-bib-0043] Tagami, S. , M. Okochi , K. Yanagida , et al. 2008. “Regulation of Notch Signaling by Dynamic Changes in the Precision of S3 Cleavage of Notch‐1.” Molecular and Cellular Biology 28: 165–176. 10.1128/MCB.00863-07. 17967888 PMC2223315

[acel70563-bib-0044] Teo, Y. V. , N. Rattanavirotkul , N. Olova , et al. 2019. “Notch Signaling Mediates Secondary Senescence.” Cell Reports 27: 997–1007.e1005. 10.1016/j.celrep.2019.03.104. 31018144 PMC6486482

[acel70563-bib-0045] Wang, D. , and X. Wang . 2022. “Diosgenin and Its Analogs: Potential Protective Agents Against Atherosclerosis.” Drug Design, Development and Therapy 16: 2305–2323. 10.2147/DDDT.S368836. 35875677 PMC9304635

[acel70563-bib-0046] Wellcome Trust Case Control, C . 2007. “Genome‐Wide Association Study of 14,000 Cases of Seven Common Diseases and 3,000 Shared Controls.” Nature 447: 661–678. 10.1038/nature05911. 17554300 PMC2719288

[acel70563-bib-0047] Wu, T. , Y. Wu , D. Jiang , et al. 2023. “SATB2, Coordinated With CUX1, Regulates IL‐1beta‐Induced Senescence‐Like Phenotype in Endothelial Cells by Fine‐Tuning the Atherosclerosis‐Associated p16(INK4a) Expression.” Aging Cell 22: e13765. 10.1111/acel.13765. 36633253 PMC9924951

[acel70563-bib-0048] Wu, Y. , D. Jiang , Q. Liu , et al. 2024. “Cathepsin L Induces Cellular Senescence by Upregulating CUX1 and p16(INK4a).” Aging (Albany NY) 16: 10749–10764. 10.18632/aging.205955. 38944813 PMC11272106

[acel70563-bib-0049] Yan, S. , L. Lu , Y. Wu , et al. 2025. “PCBP2 Regulates p16(INK4a)‐Dependent Cellular Senescence in Response to Iron.” Aging Cell 24: e70283. 10.1111/acel.70283. 41216990 PMC12686567

[acel70563-bib-0050] Yue, X. , H. Jiang , Y. Xu , M. Xia , and X. W. Cheng . 2020. “Cathepsin K Deficiency Impaired Ischemia‐Induced Neovascularization in Aged Mice.” Stem Cells International 2020: 6938620. 10.1155/2020/6938620. 32676120 PMC7346230

[acel70563-bib-0051] Zhang, L. , L. E. Pitcher , M. J. Yousefzadeh , L. J. Niedernhofer , P. D. Robbins , and Y. Zhu . 2022. “Cellular Senescence: A Key Therapeutic Target in Aging and Diseases.” Journal of Clinical Investigation 132: e158450. 10.1172/JCI158450. 35912854 PMC9337830

