# Supplementary material for: Ligand‐Independent Activation of Notch1 by Cathepsin L Induces CUX1/p16INK4a ‐Dependent Endothelial Senescence Associated With Atherosclerosis
Source: Aging Cell. 2026 May 28;25(6):e70563. doi: 10.1111/acel.70563 (PMC13239849; doi:10.1111/acel.70563)
Supplement: Supplementary file 1 — Appendix S1: acel70563‐sup‐0001‐AppendixS1.docx. [file ACEL-25-e70563-s001.docx]

**Supplementary figure 1. Western blots showing no change of H3CS1, H3 and sirt1 expression in the p10 ECs treated by Z-FY-CHO, a CTSL specific inhibitor.**


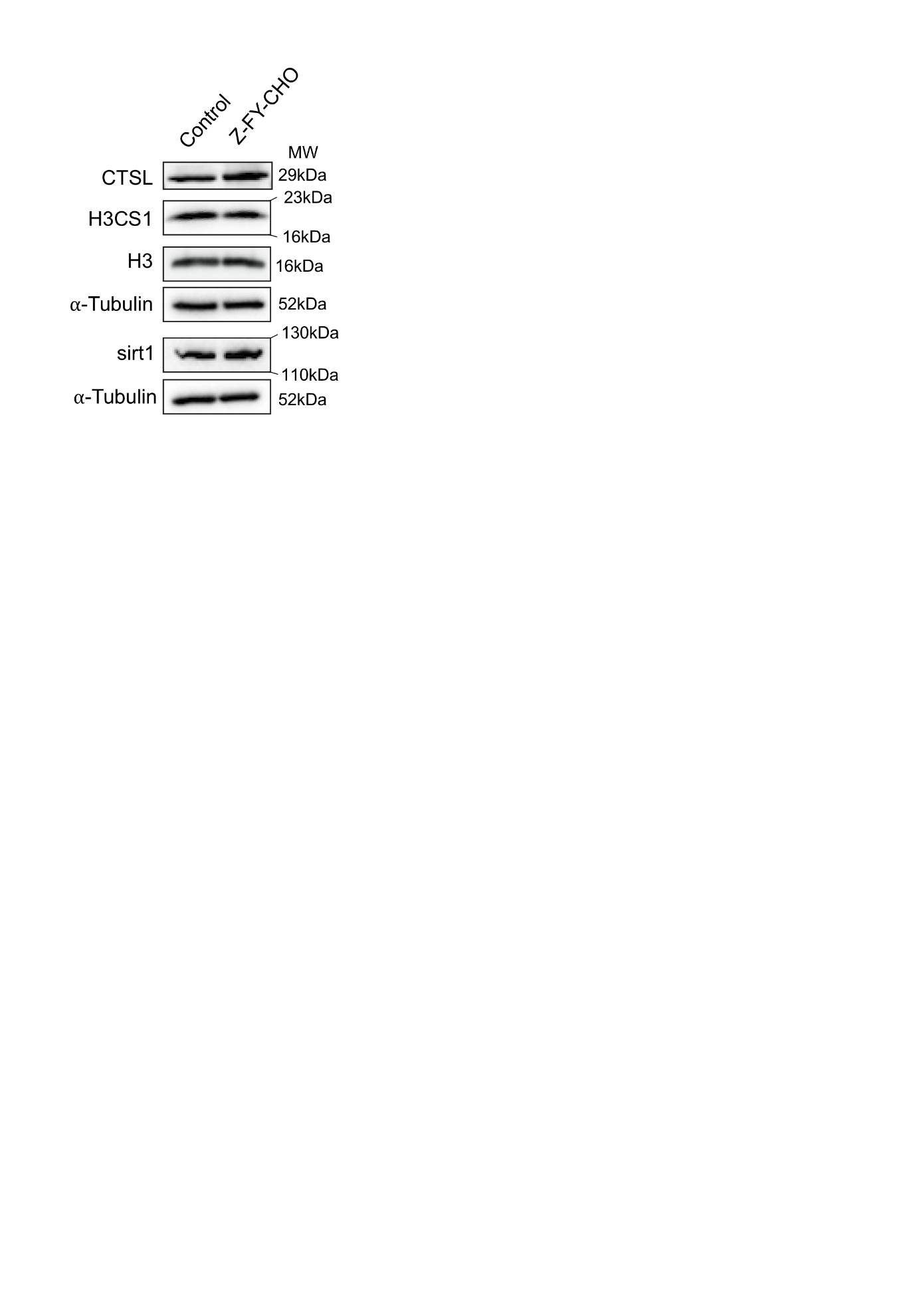


**Supplementary figure 2. Overexpression of NICD restores cellular senescence downregulated in the shRNA-mediated CTSL knockdown EC. A.** Western blots showing that overexpression of NICD (OE-NICD) restores CUX1 and p16^INK4a^ expression in the shRNA-mediated CTSL knockdown (shCTSL) p10 human ECs. **B.** SA-β-gal and γ-H2AX staining demonstrating that the decreased cellular senescence generated by shRNA-mediated CTSL knockdown can be rescued by overexpression of NICD in shRNA-mediated CTSL knockdown human ECs. Data for Western blots represent three biologically independent samples (n=3). Quantitative plots for both β-gal^+^ cells (%) in SA-β-gal staining and γ-H2AX foci/cells (%) with γ-H2AX staining are shown on the right of the panel.

**
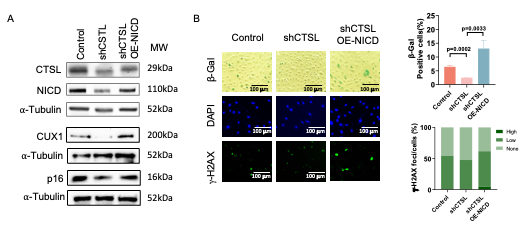
**

**Supplementary figure 3. Overexpression of RBPJ restores cellular senescence downregulated in the shRNA-mediated CTSL knockdown EC. A**. Western blots showing that overexpression of RBPJ (pLVX-RBPJ) restores CUX1 and p16^INK4a^ expression in the shRNA-mediated CTSL knockdown (shCTSL) p10 human ECs. **B**. SA-β-gal and γ-H2AX staining showing that overexpression of RBPJ (pLVX-RBPJ) restores cellular senescence in the shRNA-mediated CTSL knockdown (shCTSL) human ECs. Quantitative plots for both β-gal^+^ cells (%) in SA-β-gal staining and γ-H2AX foci/cells (%) with γ-H2AX staining are shown on the right of the panel.


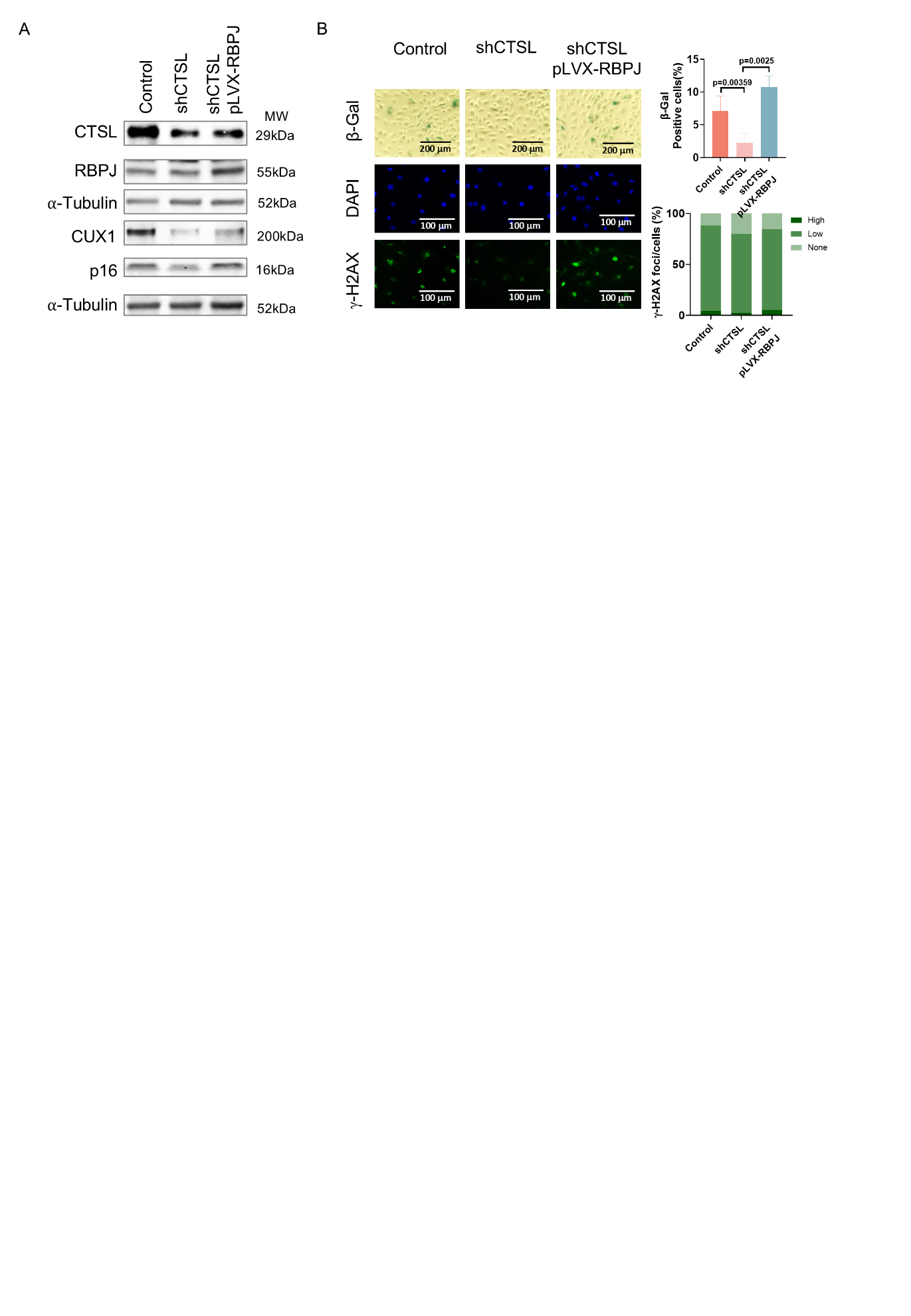


**Supplementary figure 4. Downregulation of CUX1 in mouse aortic ECs (MAECs) suppresses senescence by inhibiting p16^INK4a^. A.** and **B.** Western blot and qPCR analyses showing that siRNA-mediated CUX1 knockdown (siCUX1) downregulates p16^INK4a^ in p6 MAECs. **C**. β-Gal and γ-H2AX and staining showing a decreased cellular senescence in the siRNA-mediated CUX1 knockdown MAECs. Quantitative plots for both β-gal^+^ cells (%) in SA-β-gal staining and γ-H2AX foci/cells (%) with γ-H2AX staining are shown in the lower panel. **D**. Reduced expression of the SASP genes *IL-6*, *ICAM-1*, and *VCAM-1* demonstrating a decreased cellular senescence in the siRNA-mediated CUX1 knockdown MAECs. **E**. BrdU incorporation assay showing that siRNA-mediated CUX1 knockdown (siCUX1) induces cell proliferation. Data for Western blot analysis represent three (n = 3) independent biological samples. Data for qPCR analysis represent a combination of three (n = 3) independent biological repeats. Data for BrdU represent a combination of six (*n* = 6) biologically independent experiments. * p < 0.05; ** p < 0.01; *** p < 0.001; **** p < 0.0001. Of note, even though the human CUX1-binding sequence that contains the atherosclerosis-associated fSNP rs1537371 on the *CDKN2A/B* locus is not conserved in mice, we demonstrated that the function of CUX1 is conserved between humans and mice, as downregulation of CUX1 in mouse aortic endothelial cells (MAECs) also results in an inactivation of p16^INK4a^ expression and suppression of cellular senescence. Here, it is worth to mention that while DNA sequence conservation is important, function conservation is generally considered more important as it directly reflects the biological role of a gene.

**
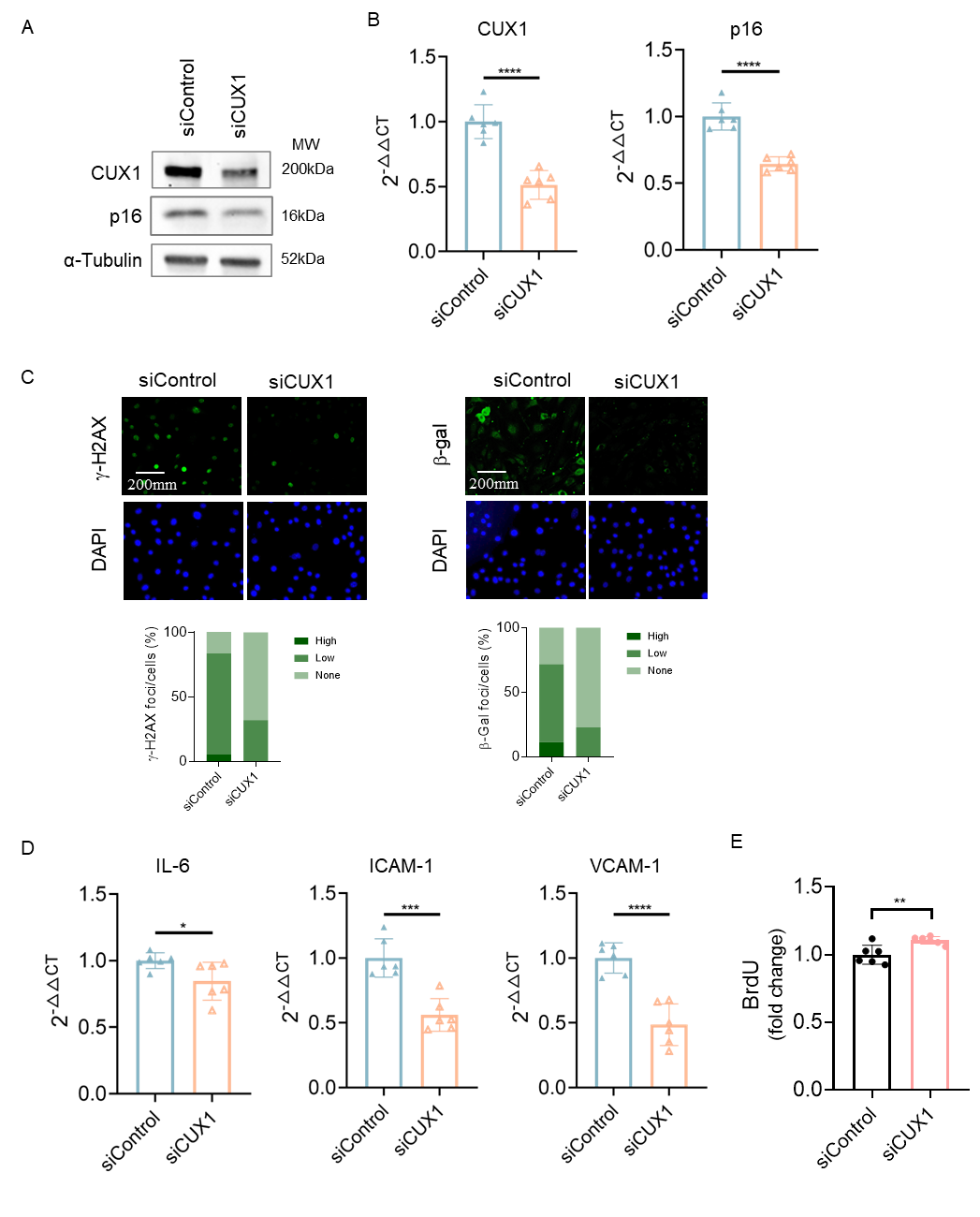
**

**Supplementary figure 5. Strategy and genotyping of the CUX1-FLX mice. A.** Simplified scheme showing the wild-type allele (w) (upper) and the CUX1 exon 6 floxed allele (f) (lower) before and after Cre recombination. Deletion of exon 6 results in an alternative splicing between exon 5 and 7, which generates a premature stop codon in exon 8. **B.** DNA gel images for genotyping the 5’ floxP insert using F1 and R1 primers. A 115 bp fragment indicates the insertion of floxP, and a 75 bp fragment indicates the wild-type allele. **C**. DNA gel images for genotyping the 3’ floxP insert using F2 and R2 primers. A 153 bp fragment indicates the insertion of floxP, and a 113 bp fragment indicates the wild-type allele. M: DNA marker. **D**. Western blots showing low level of a truncated protein with an apparent molecular weight of 34 kDa. This truncated CUX1 is unlikely functional since all the functional domains in the CUX1 protein, including four DNA binding domains (Cut repeats 1 to 3 and the homeodomain) and the two active repression domains, are deleted.

**
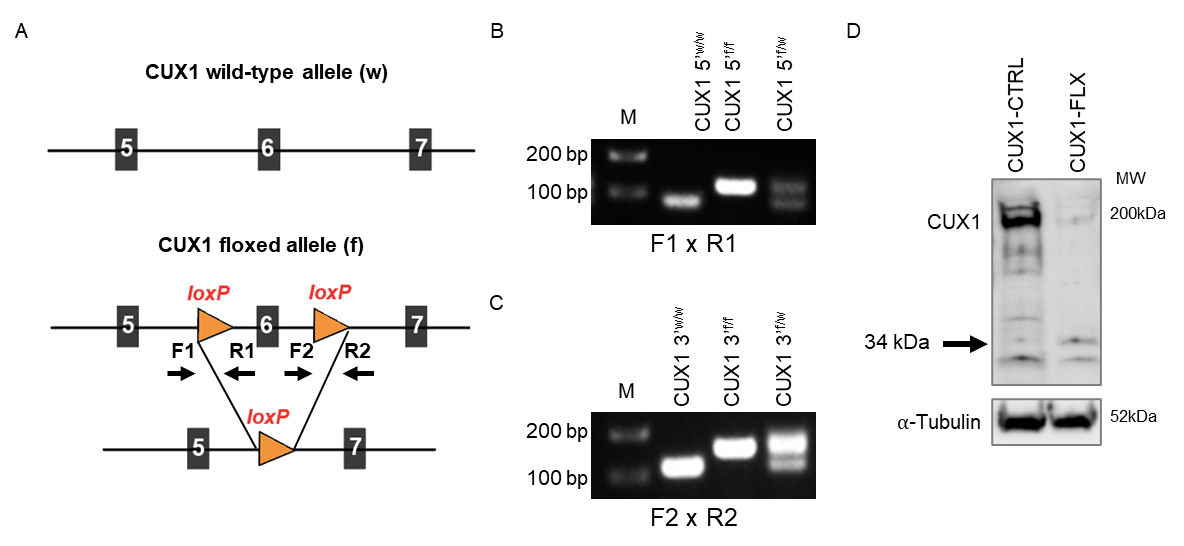
**

**Supplementary figure 6. A. Immunostaining of SA-β-gal and γ-H2AX in the whole plaques**. Immunostaining showing suppression of SA-β-gal in the whole plaques of the CUX1-FLX mice (n = 4) (**right**) versus the CUX1-CTRL mice (n = 4) (**left**). **B.** Immunostaining showing suppression of γ-H2AX in the whole plaques of the CUX1-FLX mice (n = 4) (**right**) versus the CUX1-CTRL mice (n = 4) (**left**). In both A and B, edge staining effects are observed.

**Supplementary figure 7. Densitometry analysis for Fig. 1A-1E; Fig. 2A, 2B, and 2F; Fig. 3A, 3B and 3E; Fig. 4C and Fig. 5A.**

**
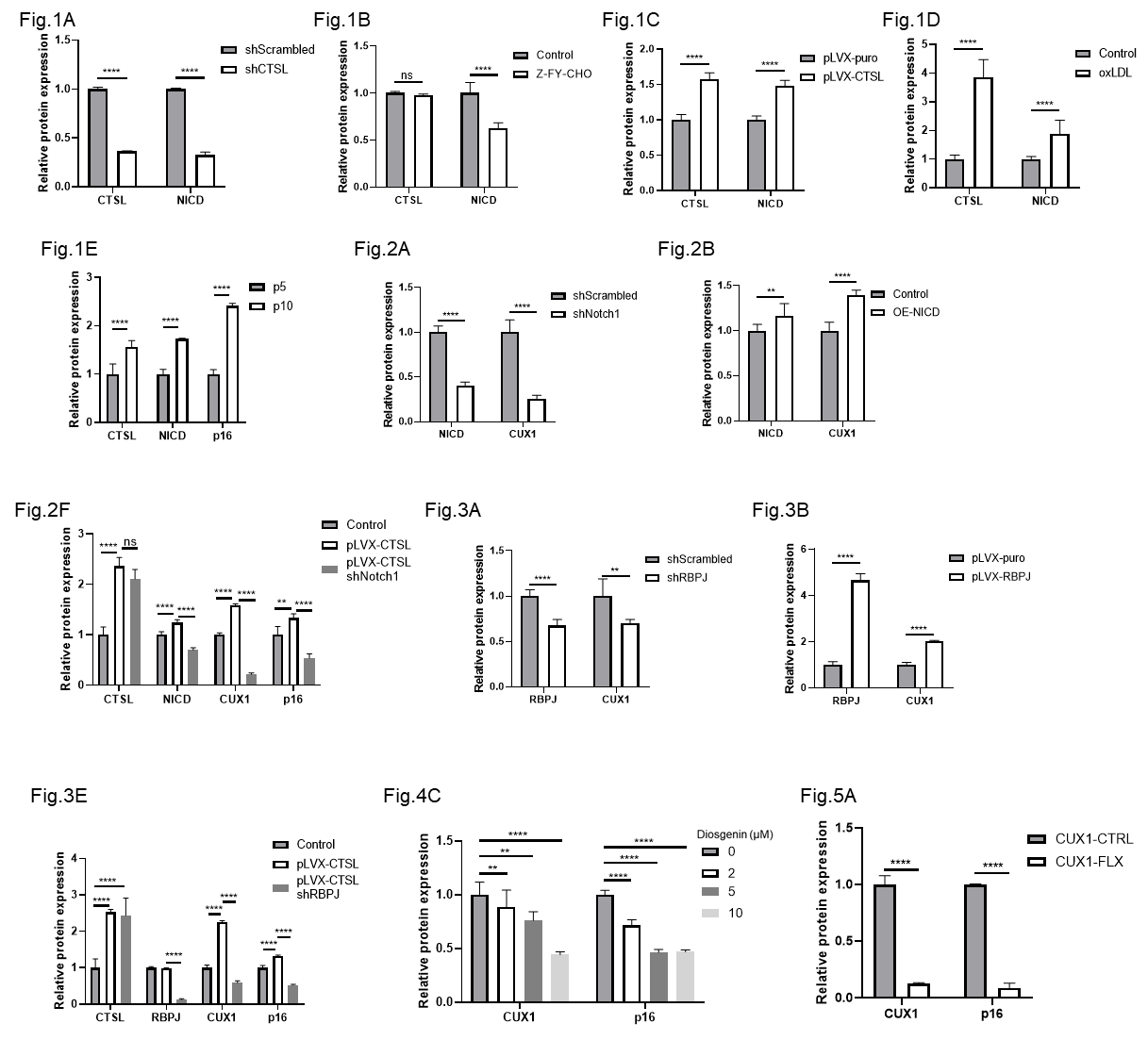
**

**Supplementary Table 1**. **Primers used in this paper**

| Usage | Primer name | Sequence |
| --- | --- | --- |
|  | CUX1-F | CCATGGAGTTTGCACCGT |
|  | CUX1-R | CAGCGAGCGGTTCTTCTC |
|  | RBPJ-F | TCATGCCAGTTCACAGCAGTGG |
|  | RBPJ-R | TGGATGTAGCCATCTCGGACTG |
|  | IL-6-F | GCAGAAAACAACCTGAACCTT |
|  | IL-6-R | ACCTCAAACTCCAAAAGACCA |
|  | ICAM1-F | AGCGGCTGACGTGTGCAGTAAT |
|  | ICAM1-R | TCTGAGACCTCTGGCTTCGTCA |
|  | IL1β-F | ACAGATGAAGTGCTCCTTCCA |
|  | IL1β-R | GTCGGAGATTCGTAGCTGGAT |
|  | GAPDH-F | CGACCACTTTGTCAAGCTCA |
|  | GAPDH-R | AGGGGTCTACATGGCAACTG |
|  | mouse ICAM-1 F | AAACCAGACCCTGGAACTGCAC |
|  | mouse ICAM-1 R | GCCTGGCATTTCAGAGTCTGCT |
|  | mouse p16 F | TGTTGAGGCTAGAGAGGATCTTG |
|  | mouse p16 R | CGAATCTGCACCGTAGTTGAGC |
|  | mouse CUX1 F | TCGAGGAACTTGCCACAC |
|  | mouse CUX1 R | TGAACAGTTCTCTGCGGATC |
|  | mouse IL-6 F | TACCACTTCACAAGTCGGAGGC |
|  | mouse IL-6 R | CTGCAAGTGCATCATCGTTGTTC |
|  | mouse VCAM-1 F | GCTATGAGGATGGAAGACTCTGG |
|  | mouse VCAM-1 R | ACTTGTGCAGCCACCTGAGATC |
|  | mouse β-actin F | CATTGCTGACAGGATGCAGAAGG |
|  | mouse β-actin R | TGCTGGAAGGTGGACAGTGAGG |
| mouse genotype | CUX1 lox F1 | TATGGAGCCCTCATCCACTAA |
|  | CUX1 lox R1 | GGAAACGACCGTCGAGTTTA |
|  | CUX1 lox F2 | CTCAGATACAGCTCAGCGTAGA |
|  | CUX1 lox R2 | GGGACAAGGATCACTGTCATTT |
|  | CRE-F | ATGTCCAATTTACTGACCGTACACC |
|  | CRE-R | CACCGTCAGTACGTGAGATATC |
|  | APOE_M_F | GCCTAGCCGAGGGAGAGCCG |
|  | APOE_M_R-WT | TGTGACTTGGGAGCTCTGCAGC |
|  | APOE_M_R-MUT | GCCGCCCCGACTGCATCT |
| shRNA knockdown | CUX1 shRNA | GCACGATATTGAAACAGAGAA |
|  | CTSL shRNA | ACTGGTAAATGTTACCTCTA |
|  | RBPJ shRNA | CCGGGCATGTAGAAGGAGGTAATTTCTCGAGAAATTACCTCCTTCTACATGCTTTTTG |

**Supplementary Table 2. Antibodies used in this paper**

| antibody | Manufacturer | Cat# | Usage |
| --- | --- | --- | --- |
| CTSL | proteintech | 27952-1-AP | WB |
| CTSL | Invitrogen | PA5-100424 | IF |
| CUX1 | abclonal | A2213 | WB, IF |
| α-tubulin | thermofisher | T9026 | WB |
| p16^INK4a^ | proteintech | 10883-1-AP | WB |
| γ-H2AX | Santa Cruz | sc-517348 | IF |
| mouse p16^INK4a^ | Thermofisher | PA5-20379 | WB, IF |
| ICAM-1 | Thermofisher | MA5407 | IF |
| β-Gal | proteintech | 15518-1-AP | IF |
| RBPJ | active motif | 61505 | IF |
| Goat anti-Mouse IgG (H+L) Fluor 488-conjugated antibody | thermofisher | A-11001 | IF |
| Donkey anti-Sheep IgG (H+L) 594 conjugated antibody | thermofisher | A-11016 | IF |
| Goat anti-Rat IgG (H+L) 594 conjugated antibody | thermofisher | A-11007 | IF |
| Goat anti-Rabbit IgG (H+L) 594 conjugated antibody | thermofisher | A-11012 | IF |
| Donkey anti-Goat IgG (H+L) 594 conjugated antibody | Thermofisher | A-11058 | IF |
